# Genome-Wide Identification and Characterization of the Polyamine Uptake Transporter (Put) Gene Family in Tomatoes and the Role of Put2 in Response to Salt Stress

**DOI:** 10.3390/antiox12020228

**Published:** 2023-01-18

**Authors:** Min Zhong, Lingqi Yue, Wei Liu, Hongyi Qin, Bingfu Lei, Riming Huang, Xian Yang, Yunyan Kang

**Affiliations:** 1 College of Horticulture, South China Agricultural University, Guangzhou 510642, China; 2 Key Laboratory for Biobased Materials and Energy of Ministry of Education, Guangdong Provincial Engineering Technology Research Center for Optical Agriculture, College of Materials and Energy, South China Agricultural University, Guangzhou 510642, China; 3 College of Food Science, South China Agricultural University, Guangzhou 510642, China

**Keywords:** polyamine uptake protein, Put2, antioxidants, reactive oxygen species, salt stress, tomato

## Abstract

The polyamine uptake transporter (Put), an important polyamines-related protein, is involved in plant cell growth, developmental processes, and abiotic stimuli, but no research on the Put family has been carried out in the tomato. Herein, eight tomato Put were identified and scattered across four chromosomes, which were classified into three primary groups by phylogenetic analysis. Protein domains and gene structural organization also showed a significant degree of similarity, and the *Put* genes were significantly induced by various hormones and polyamines. Tissue-specific expression analysis indicated that *Put* genes were expressed in all tissues of the tomato. The majority of *Put* genes were induced by different abiotic stresses. Furthermore, *Put2* transcription was found to be responsive to salt stress, and overexpression of *Put2* in yeast conferred salinity tolerance and polyamine uptake. Moreover, overexpression of *Put2* in tomatoes promoted salinity tolerance accompanied by a decrease in the Na^+^/K^+^ ratio, restricting the generation of reactive oxygen and increasing polyamine metabolism and catabolism, antioxidant enzyme activity (SOD, CAT, APX, and POD), and nonenzymatic antioxidant activity (GSH/GSSG and ASA/DHA ratios, GABA, and flavonoid content); loss of function of *put2* produced opposite effects. These findings highlight that Put2 plays a pivotal role in mediating polyamine synthesis and catabolism, and the antioxidant capacity in tomatoes, providing a valuable gene for salinity tolerance in plants.

## 1. Introduction

Polyamines, one of the organic polycations, are abundant in various plant organisms and are involved in various cellular processes such as cell growth, nucleic acid stability, and protein synthesis [1,2]. The most abundant polyamines in plant cells, diamine putrescine, triamine spermidine, and tetraamine spermine, are strongly associated with plant responses to biotic and abiotic cues [3,4]. Manipulation of putrescine, spermidine, and spermine by chemical or genetic means is essential for many developmental processes [5,6]. The crucial roles of polyamine synthesis and metabolism in response to numerous stresses have been demonstrated by genetic manipulation [7]. Intracellular polyamine pools are critical for the intermediary part of nitrogen metabolism, and also crosstalk with other metabolic pathways, such as hormones, small molecule signals, and stress-response complexes [8,9].

Besides, increasing evidence indicates that the uptake of polyamine plays essential functions in coordinating the response of plants to a variety of environmental stresses. A salt-sensitive cultivar of rice supplied with putrescine in roots exhibited an increased grain yield [10]. The *arginine decarboxylase* (*adc*) mutant in *Arabidopsis* showed hypersensitivity to low-temperature stress, but the tolerance was enhanced after being fed putrescine in *adc* mutants [11]. These studies show that polyamine transport could be an important component of diverse environmental protection. In *Arabidopsis*, methyl viologen 1 (At5g05630, *AtRMV1*), a L-type amino acid transporter, was recently found to be a protein important for paraquat (PQ) and uptake of polyamine [12]. The *Arabidopsis* mutant (AT1G31830, *pqr2*/*AtPut2*) also encodes a polyamine transporter and negatively responds to ABA signaling [13,14]. The protective influences of polyamine in opposition to PQ toxicity are partly attributed to transport interactions between polyamines and PQ because both have similar uptake characteristics. In addition, the *paraquat resistant1 (pr1)* mutant exhibited inefficient absorption of PQ [13]. The *put5* plants produced fewer flowers, flowered earlier, and had smaller leaves than wild-type (WT) plants, while the *OsPut1* or *OsPut3* over-expression plants showed a buildup of spermidine and conjugated-spermidine in leaves, larger leaves, more flowers, and a delay in the flowering time, which is implicated in polyamine transport [15]. The rice *OsPut1/2/3* mutant was created by CRISPR/Cas9 gene editing, and the *OsPut1/2/3* mutant increases PQ tolerance without significant yield loss [16]. Moreover, *OsPut1*-*OsPut3* was shown to have a high affinity for spermidine uptake through the substrate assay with a yeast polyamine uptake mutant (*agp2*Δ), and *AtPut1*-*AtPut3* has similar properties [17]. However, much less attention has been paid to transport proteins in plants. To our knowledge, very little has been unraveled regarding the tomato polyamine uptake protein (Put) family and their functions in abiotic stress.

The tomato is one of the world’s most significant cash crops and is sensitive to biotic and abiotic pressures such as salt stress, low and high temperatures, and so on. These unfavorable environmental factors seriously compromise tomato growth and yield. Under stressful conditions, polyamine anabolism and catabolism have been found to have important roles involving a multitude of mechanisms [18]. For example, polyamine oxidase, arginine decarboxylase, *S*-adenosylmethione synthetase, and spermine synthase act as critical mediators in multiple stress conditions [19,20,21,22,23]. To date, it has been widely accepted that polyamine acts as a crucial antioxidant in plants [24]. The increase of endogenous polyamines levels saves cells by eliminating reactive oxygen species (ROS) and boosting the antioxidant capacity in response to oxidative stress [25]. Nevertheless, the implications of Put in response to environmental stresses remains elusive. In particular, the role of Put in polyamine anabolism and catabolism, as well as in antioxidant activity, remains largely unknown. In this study, by identifying and characterizing members of the Put gene family in the tomato, a family known for polyamines uptake, we unraveled that Put2, a candidate of the Put family, had a favorable function in salt tolerance via modulating polyamine metabolism and antioxidants. This study sheds fresh light on the important role of Put-mediated polyamine homeostasis in tomatoes, as well as its significance for plant fitness.

## 2. Material and Methods

### 2.1. Indentation and Sequence Analysis of the Tomato Put Family

The protein sequence of tomato Put and *Arabidopsis* AtPut were downloaded from the NCBI (http://www.ncbi.nlm.nih.gov/, accessed on 10 January 2022). The SGN database (https://solgenomics.net/, accessed on 10 January 2022) was used to get the tomato (SL4.0) reference genome sequence and annotations. We identified eight putative Put proteins encoded in the tomato genome based on investigations of the Arabidopsis Put protein. The eight Put genes in tomato were called by their chromosomal locations. Expasy, an online software, was used to gather basic information for all Put proteins, including their molecular weight (Mw) and isoelectric point (pI).

### 2.2. Alignment of the Protein Sequence and Phylogenetic Tree Construction

Using MEGA X, the protein sequences of Put in tomato, rice, and *Arabidopsis* were aligned [26]. Poor alignment areas from all protein sequences were removed using the trimAl tool, and a phylogenetic tree was created using the maximum-likelihood (ML) technique with the Poisson correction and 1000 bootstrap repetitions in IQ-TREE [27]. The depiction of the phylogenetic tree was constructed by Evolview (www.evolgenius.info/, accessed on 22 January 2022). A phylogenetic tree of the tomato Put protein was also built independently. The Put protein sequences are provided in Supplemental data S1.

### 2.3. Analyses of Conserved Motifs, Conserved Domains, Cis-Acting Elements in Promoters, and miRNA Prediction

The conserved motifs of the tomato Put proteins were performed by the MEME tool (5.05) (http://meme.nbcr.net/meme/, accessed on 22 January 2022), and Pfam (http://pfam.xfam.org/, accessed on 22 January 2022) was used to predict the conserved domains of the tomato Puts proteins. The promoter regions of Put were created using the 2.0 kb genomic DNA sequence upstream of the translation start codon (ATG). PlantCare took the cis elements from the Put promoter regions. In Supplemental data S2, the cis elements are listed. The relevant data visualization was conducted using TBtools. According to the targeted candidate, as described previously, the Put coding sequences were submitted to the psRNATarget serve to predict the miRNAs (https://www.zhaolab.org/psRNATarget/, accessed on 22 January 2022) [28]. The Puts protein’s transmembrane domains were predicted using the TMHMM program [29].

### 2.4. Plant Material and Treatments

Three-week-old tomato (*Solanum lycopersicum* L. cv. Ailsa Craig) seedlings were treated with different exogenous polyamines, hormones, and oxidative stress, or abiotic stresses. Briefly, 2.0 mM Put, 1.0 mM Spd, or 2.0 mM Spm were sprayed over the seedlings; for hormone treatments and oxidative stress, 100 μM ABA, 2 mM SA, 100 μM GA_3_, 40% ethylene (ETH), 100 μM paraquat, and water were also sprayed onto the tomato plants, respectively. For RNA extraction, at 0, 30 min, 1, 3, 6, and 12 h, samples of leaves were taken, accordingly. The control was the water treatment at 0 h.

We then investigated Put genes response to different abiotic stresses. Salt and drought stress were initiated by irrigating the plants with 200 mM NaCl or 20% PEG6000 solution, respectively. Tomato seedlings were subjected to 42 °C (high temperature), and 4 °C (low temperature) for heat and cold stress, respectively. After, the treated samples were respectively collected after 0, 30 min, 1, 3, 6, and 12 h; the samples at 0 h were used as the control. In addition, tissues (root, stem, leaf, bud, flower, and fruit) were harvested for investigation of tissue-specific expression. After each treatment, leaves from different plants (three biological replicates) were quickly frozen in liquid nitrogen and kept at −80 °C for further analysis.

### 2.5. Yeast Strain and Culture Conditions

The wild type (WT), G19 (Δ*ena1–4*), failure to mediate Na^+^ uptake; CY162, the K^+^ uptake-deficient, and a yeast strain impaired in spermidine uptake, *agp2*Δ (strains obtained from open biosystems, http://www.openbiosystems.com/GeneExpression/Yeast/ORF/, accessed on 10 May 2022), were used to describe the potential transporter. The yeasts were grown in YPD media at 28 °C. The yeast cells were converted using lithium acetate. Put1-8 coding sequences were respectively cloned into the pYES2 expression vector.

Wild type-empty vector and *agp2*Δ-Put1-8 transformants were grown in yeast extract peptone galactose (YPG) medium. Cell suspensions were serially diluted as OD_600_ of 0.6 for growth tests, and 5 μL aliquots were spotted onto YPG plates containing 25 mM spermidine and 1.5 mM paraquat. After 3–4 days of incubation at 28 °C, the plates were photographed. For polyamine transport assays, the yeast cells were harvested at the mid-logarithmic phase, washed with ddH_2_O, and suspended in the YPG media at a dose of 10^8^ cells/mL. One-hundred-microliter aliquots cells were transferred to the Eppendorf tubes, and polyamine absorption was activated by the addition of Spermidine or Putrescine at 25 μM concentrations. The absorption was inhibited by adding 1.5 mL of ice-cold uptake buffer with excessive spermidine content at selected times, filtered by a 0.45 μm membrane, and washed with 2 mL ice-cold ddH_2_O (three times) to remove the exogenous polyamines. Polyamine determination in vivo was performed by an Agilent high-performance liquid chromatography 1200 series system (HPLC, Agilent Technologies, Santa Clara, CA, USA). Polyamines were obtained from Sigma-Aldrich (St Louis, MO, USA).

For salt tolerance assays, the final pYES2-empty and pYES2-Put1-8 vectors cultured in G19 and CY162 were performed on SD-U (Synthetic Dextrose Minimal Medium without Uracil) medium, and then diluted until the OD_600_ value = 0.6. 5 μL. Aliquots were spotted onto YPG plates containing 100 mM NaCl and 0.1 mM KCl, respectively, and incubated at 28 °C. No treatment was added for the control. After 3–4 days of incubation, the plates were photographed. For Na^+^ and K^+^ uptake treatment, the empty and positive yeast were incubated to OD_600_ = 1.0, the supporting was discarded, and 50 mL ddH_2_O was used to wash the yeast. The yeast cells were obtained by centrifugation, followed by starvation treatment with AP liquid medium without K^+^ and Na^+^. After starvation treatment, the yeast cells were obtained by centrifugation and treated as follows: inoculating the yeast with an AP liquid medium including 200 μM NaCl and 200 μM KCl, respectively. Subsequently, the liquids were put on a 28 °C shaker (220 r/min), and 4 mL of bacterial solution was taken every 10 min, centrifuged, and the supernatant was collected for analysis of Na^+^ and K^+^ contents. Three biological replicates were made. The primers for vector construction are listed in Supplemental data S3 (Table S1).

### 2.6. Plasmid Construction and Plant Transformation

The *Put2*-overexpression vector (full-length coding sequence of *Put2*) was constructed as previously described [30]. Gene loss-function of *put2* lines was generated through gene editing approaches. To generate the CRISPR/Cas9 vector, the two target sequences for *put2* were designed using the online software CRISPR-GE (http://skl.scau.edu.cn/targetdesign/, accessed on 22 January 2022), which were inserted into two single guide RNA (sgRNA) expression cassettes through overlap PCR, followed by cloning into the pYLCRISPR/Cas9Pubi-H vector via Golden Gate ligation method [31]. The confirmed pFGC1008-*Put2*-3HA vector and pYLCRISPR/Cas9Pubi-H-*put2* binary vector were transformed into *Agrobacterium tumefaciens* strain GV3101 by electroporation after transgenic plants were generated with *Agrobacterium*-mediated cotyledon transformation of *Solanum lycopersicum* cv. Ailsa Craig via a method previously described [32]. Two separate homozygous T2 lines from mutation and overexpression lines were confirmed with Sanger sequencing and qRT-PCR. The *put2* mutants and *Put2*-OE plants were used in this study. The primers for vector construction are listed in Supplemental data S3 (Table S1).

### 2.7. Salt Treatment and Salt Tolerance Assays

The tomato seedlings (WT, *put2* mutants, and *Put2* overexpression lines) were used for salt tolerance experiments. After seed germination and two cotyledons full expansion, the seedlings were cultured in 250 cm^3^ plastic pots filled with a peat-vermiculite combination (2:1, *v:v*). The seedlings were placed in a greenhouse at 28 ± 2 °C/20 ± 2 °C (day/night) under a maximum photosynthetic photon flux density (PPFD) of approximately 1200 μmol m^−2^ s^−1^ and a relative humidity of 70–80%. They were watered daily using Hoagland nutrient solution. Three-week-old WT and transgenic tomatoes of uniform size and health growth status were selected and subjected to salt stress treatment. The seedlings were treated by watering the plants with 200 mL of 200 mM NaCl every other day for salt stress. The control treatment was replaced with an equal amount of water. The salt stress treatment lasted for 7 days, and pictures were captured. The maximum quantum yield of PSII (Fv/Fm) was examined with the Imaging-PAM system (IMAG-MAXI; Heinz Walz, Effeltrich, Germany), as previously described by Zhong et al. [30]. The relative electrolyte leakage (REL%), Na^+^, and K^+^ analysis was performed as described previously by Zhong et al. [30]. The plants were enclosed in envelopes and placed in an oven at 105 °C for 30 min, and then the oven temperature was adjusted to 75 °C to obtain a permanent dry weight (DW).

### 2.8. Determination of Polyamine Content

The free polyamines content was analyzed by Agilent 1200 High-performance Liquid Chromatography (HPLC, Agilent Technologies, Santa Clara, CA, USA), as previously described in Zhong et al. [33] with slight modifications. Frozen plant tissue (leaves sample, 0.5 mg) was ground with liquid nitrogen, used 10:1 (*v/w*) of extraction buffer (5% cold aqueous perchloric acid (PCA), *w/w*). Samples were incubated for 1 h at 4 °C, then centrifuged at 15,000× *g* for 10 min at 4 °C. Volumes of 200 μL of collected supernatant were mixed with 15 μL benzoyl chloride and incubated for 1 h at 60 °C in darkness conditions. Four milliliters of saturation NaCl solution was used to quench the reaction and diethyl ether was added; 5 mL cold ethyl acetate was then added to extract polyamines. Then, organic layers were evaporated to dryness, redissolved in 100 μL methanol, and filtered with a 0.45 μm pore nylon filter. A volume of 25 μL extraction solution was used to determine the endogenous polyamines levels. The mobile phase was with 64% (*v/v*) methanol and had a flow rate of 0.8 mL min^−1^. Putrescine, spermidine, spermine, and cadaverine (Sigma, St. Louis, MO 63178, USA) were chosen as standard samples and treated similarly.

### 2.9. Determination of PAO Enzymatic Activities and H_2_O_2_ Content

Amine oxidase was determined as previously described by Su et al. [34] and Urra et al. [9]. Leaf samples (0.5 g) were ground with liquid nitrogen, homogenized 2:1 (*v/w*) in 100 mM sodium phosphate buffer (pH 6.5), and centrifuged at 12,000× *g* for 20 min at 4 °C. One-hundred-microliters of the recovered supernatant was mixed with the 3 mL reaction mix, which contained 2.5 mL sodium phosphate buffer (100 mM, pH 6.5), 200 μL 15 mM 4-aminoantipyrine/0.2% (*v/v*) *N*, *N*-dimethylaniline, 100 μL 250 U ml^−1^ peroxidase, and 100 μL 20 mM putrescine as a substrate. The CuAO_Put_, PAO_Spm_, and PAO_Spd_ were determined using Putrescine, Spermine, and Spermidine as substrates, respectively. A 0.01 value of the changes in absorbance at 555 nm was assayed to one activity unit of the PAO enzyme after being incubated for 30 min at 22 °C. Control samples without polyamines were used to calculate these activities. Leaf H_2_O_2_ content was determined by specific detection kits according to the manufacturer’s instructions (Nanjing Jiancheng Bioengineering Institute, Jiangsu, China).

### 2.10. Antioxidant Assay

To assess the antioxidant enzyme activity, 0.5 g leaf samples were homogenized with 3 mL ice-cold 50 mM phosphate buffer (pH 7.8), which contained of 2 mM L-ascorbic acid, 2% (*w/v*) PVPP, and 0.2 mM EDTA. Then, the homogenates were centrifuged for 20 min at 12,000× *g*, and supernatants were collected to determine the enzyme activity. The activities of SOD, CAT, APX, and POD were assayed as previously described [30]. AsA/DHA and GSH/GSSG were measured as described by Zhong et al. [35]. The GABA content was determined by the Berthelot reaction with some modifications [36]. Leaf samples (0.5 g) were ground with methanol, centrifuged at 6000× *g* for 15 min, and the supernatant was discarded. The sediment was dissolved in 1.5 mL ddH_2_O and heated in a water bath at 50 °C for 2 h, followed by centrifugation for 15 min at 7000× *g*. A volume of 1 mL supernatant was mixed with 100 μL 2 mol L^−1^ AlCl_3_ and oscillated, and then centrifuged for 10 min at 12,000× *g*. The supernatant was added with 300 μL KOH and incubated for 5 min, then centrifuged at 12,000× *g* for 10 min. The GABA content was measured according to the following procedure: 300 μL supernatant was mixed with the reaction mix, composed of 500 μL 0.1 mol L^−1^ sodium tetraborate (pH 10.0), 400 μL 6% phenol, and 600 μL 5% sodium hypochlorite. The solution was boiled for 10 min and then placed in an ice bath for 5 min. Finally, the absorbance at 645 nm was measured after shaking the solution with 2 mL 60% ethyl alcohol. The assessment of the total flavonoid concentration was determined by Zhishen et al. [37], with some modifications. Dried leaf samples (0.5 g) were homogenized with 2 mL 80% ethanol, and then added 300 μL 20 mol L^−1^ NaNO_2_; 3 mL 1 mol L^−1^ AlCl_3_ was added after 5 min, and after 6 min, 2 mL 1 mol L^−1^ NaOH was added and mixed well. Finally, the absorbance was measured at 510 nm.

### 2.11. Analysis of Gene Expression

Total RNA was extracted with the RNAsimple Total RNA Kit (Tiangen, DP419) and reverse transcribed with the HiScript^TM^ qRT SuperMix for qPCR (+gDNA wiper) kit (Vazyme, Nanjing, China). The qPCR reaction was performed by the ABI VII7 real-time PCR system (Applied Biosystems, Waltham, MA, USA). *Actin* was used as the tomato reference gene. The qRT-PCR primers are listed in Supplemental data S3 (Table S2).

### 2.12. Statistics

The data are presented as the means ± SDs and were analyzed using SPSS 20 statistical software. The experimental data were analyzed with Duncan’s multiple range test at *p* < 0.05.

## 3. Results

### 3.1. Identification of Tomato Put Family Genes

To analyze Put proteins, a query search against the tomato genome database was accomplished using *Arabidopsis* and rice Put protein as the control search (Table 1). Eight potential *Put* proteins with high sequence similarity to *AtPut* and *OsPut* were identified and called Put1-8. They had CDs sizes ranging from 1077 bp (Put6) to 1605 bp (Put3), with polypeptides of 359–535 amino acids. The theoretical isoelectric points (pI) of *Put* varied from 5.41 (*Put3*) to 9.37 (*Put8*). The molecular weights (MW) of *Put* ranged from 40.28 (*Put6*) to 58.8 (*Put3*) (Table 1). Additionally, all Puts were predicted to contain transmembrane domains (Figure S1).

### 3.2. Analysis of the Phylogenetic, Chromosomal Distribution, Gene Structure, and Promoter Sequences of Tomato Family Members

**Figure 1:**
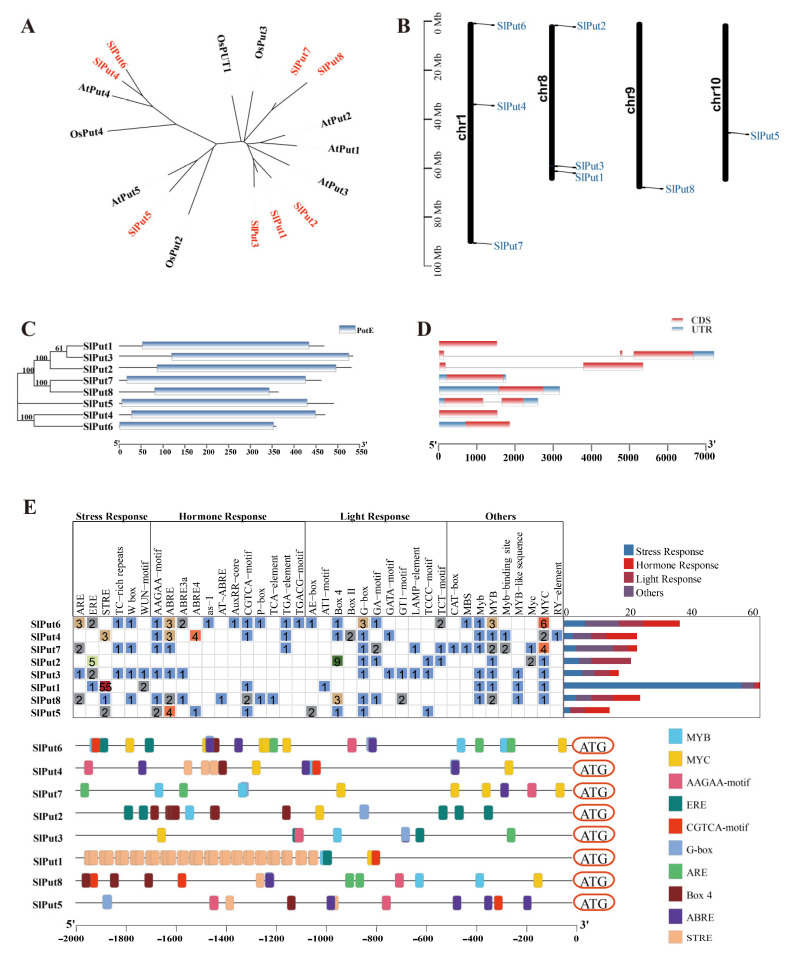
The characteristics of Put genes in the tomato. (**A**) Phylogenetic relationships of Put genes in Arabidopsis, rice, and tomato. (**B**) Gene distribution within the tomato chromosomes. The chromosome numbers are indicated on the left, and the position marked in the chromosome indicates the location of Put genes. (**C**) The protein motifs and phylogenetic trees in the Put famous members. (**D**) Gene structure of the Put family members in tomatoes. (**E**) Cis element distribution of the Put genes.

To better study the evolutionary relationships among the tomato Put protein sequences and those of other plants, a phylogenetic tree was performed through the Neighbour-Joining method. By comparing the protein sequences of the Put genes, three main groupings could be distinguished. Two *Put* genes, *Put4* and *Put6,* were included in Group I; Group II only included *Put5*, and the remaining five genes (*Put1*, *Put2*, *Put3*, *Put7,* and *Put8*) were classified into Group III (Figure 1A). According to their location, the chromosomal positions of the Put genes were analyzed. Among these *Put* genes, three (*Put4*, *Put6* and *Put7*) were mapped to chromosome 1, and three (*Put1*, *Put2* and *Put3*) were located on chromosome 8, while *Put5* and *Put8* were located in chromosomes 9 and 10, respectively. These results suggest an uneven distribution of these genes on chromosomes (Figure 1B).

To better analyze the evolutionary relationships in *Put* genes, the phylogenetic tree was also constructed. *Put1*, *Put2,* and *Put3* were observed to have a close evolutionary relationship. A close evolutionary relationship was also found in *Put7* and *Put8*, and *Put4* and *Put6*, respectively. Interestingly, *Put5* has a separate evolutionary branch. In addition, their structural domains are highly conserved and contain the polyamine transport protein PotE structural domain (Figure 1C). The Put family gene structure analysis was carried out to learn more about their intron/exon structures. One intron was present in *Put2* and *Put5*, two in *Put3*, while the other five (*Put1*, *Put7*, *Put8*, *Put4,* and *Put6*) were intron deletion genes (Figure 1D). To investigate the feasible roles of Put family genes in different abiotic stress and developmental steps, we used the PlantCARE database to estimate the presence of *cis*-acting elements in the promoter regions of the Put family. We obtained *cis*-acting elements that are associated with stress, hormone, and light responsiveness. Surprisingly, we only found five typical ethylene responsive motifs (ERE, GCC-box motifs) in the promoter of Put2, but not in that of other Puts (Figure 1E).

miRNAs are an important kind of non-coding signal strand RNAs of around 22 nucleotides that are encoded by genes in the organism and are closely involved in the regulation of genes in answer to various life processes and stresses. A total of 29 miRNAs targeting the polyamine transporter genes in the tomato are listed in Table 2, including three sly-miRNAs targeting Put1 (sly-miR390a-5p, sly-miR390b-5p, and sly-miR6022); sly-miR6024 and sly-miR6026 targeting Put2; sly-miR6023 and sly-miR1916 targeting Put3; six sly-miRNAs targeting Put4 (sly-miR9479-3p, sly-miR6024, sly-miR171c, sly-miR171a, sly-miR9472-5p, and sly-miR9478-3p); sly-miR164a-5p and sly-miR164b-5p targeting Put5; Put6 was targeted by sly-miR1917; six sly-miRNAs targeting Put7 (sly-miR156a, sly-miR156b, sly-miR156c, sly-miR390a-5p, sly-miR396a-3p, and sly-miR6023); and three sly-miRNAs targeting Put8 (sly-miR156a, sly-miR156b, sly-miR156c, sly-miR319b, sly-miR319c-3p, sly-miR396a-3p, and sly-miR6023) (Table 2).

### 3.3. Responsiveness of Put Gene Expression under Hormone, Polyamine, and PQ Treatment

**Figure 2:**
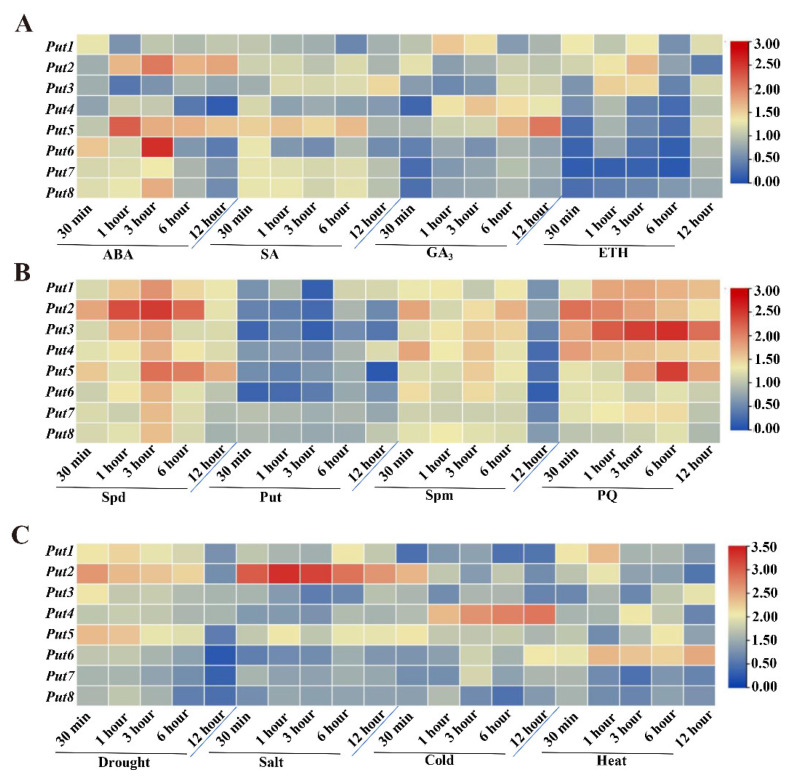
Expression patterns of *Put* genes in tomatoes under hormone, polyamine, paraquat, or abiotic stress. (**A**) Heatmap representation of the responsiveness of *Put* genes after treatment with ABA, SA, GA_3_, and ET. The scale (0 to 3) represents the expression level (from low to high). (**B**) Heatmap representation of the responsiveness of *Put* genes after treatment with Spd, Put, Spm, and PQ. The scale (0 to 3) represents the expression level (from low to high). (**C**) Heatmap representation of the responsiveness of *Put* genes after drought, salt, cold, and heat treatment. The scale (0 to 3.5) represents the expression level (from low to high). qRT-PCR was conducted after treatment, according to the methods section. The expression of WT at 0 min in the different treatments was set to 1.

The hormones in the plant kingdom play crucial roles in plant growth and development. Therefore, four different hormone-induced Put transcript-level changes were analyzed. After ABA treatment, the expression of all Puts was induced at 30 min, while that of *Put2* and *Put5* was induced after 6 h (Figure 2A). The expression of all *Puts* was induced at 30 min after SA treatment, which were also upregulated in the later time points, except for *Put*4 and *Put6* (Figure 2A). In contrast, the transcripts of *Put6*, *Put7,* and *Put8* were reduced initially following GA_3_ and ETH treatments, and ETH also reduced the *Put4* and *Put5* expression, a GA_3_ induced both. *Put2* and *Put3* were reduced after GA_3_ treatment, and then induced after 6 h of treatment, Put1 was induced after GA_3_ and ETH treatments (Figure 2A).

To examine the possible roles of *Put* genes in the tomato, transcripts of *Puts* after three treatments with polyamines, including Spermidine, Putrescine, and Spermine, were analyzed. Exogenous Spermidine treatment induced all *Put* genes, and *Put2* was the most obvious, followed by *Put5* (Figure 2B). However, the expression of all *Put* genes was reduced by Putrescine, and the reduction magnitude differed among them. After the seedlings were treated with Spermine, all *Put* genes were upregulated, but reduced after 12 h treatment. Additionally, exogenous PQ treatment caused upregulation of all Put genes similar to that of Spermidine treatment (Figure 2B).

### 3.4. Differential Expression of Put Genes during Abiotic Stress

Upon exposure to drought stress, the mRNA transcript of *Put1–5* was rapidly induced, and *Put2* was the most significant, but other members were reduced (Figure 2C). Salinity stress resulted in the downregulation of *Put3*, *Put4*, *Put6*, *Put7,* and *Put8*, and upregulation of *Put1*, *Put2,* and *Put5*. Cold stress resulted in the most pronounced induction of *Put4*. Furthermore, when the seedlings were submitted to heat stress, sustained and stable upregulation of *Put6* was observed, whereas two genes (*Put7* and *Put8*) were reduced post-treatment (Figure 2C).

### 3.5. Expression Analysis of Put Genes in Different Tissues

**Figure 3:**
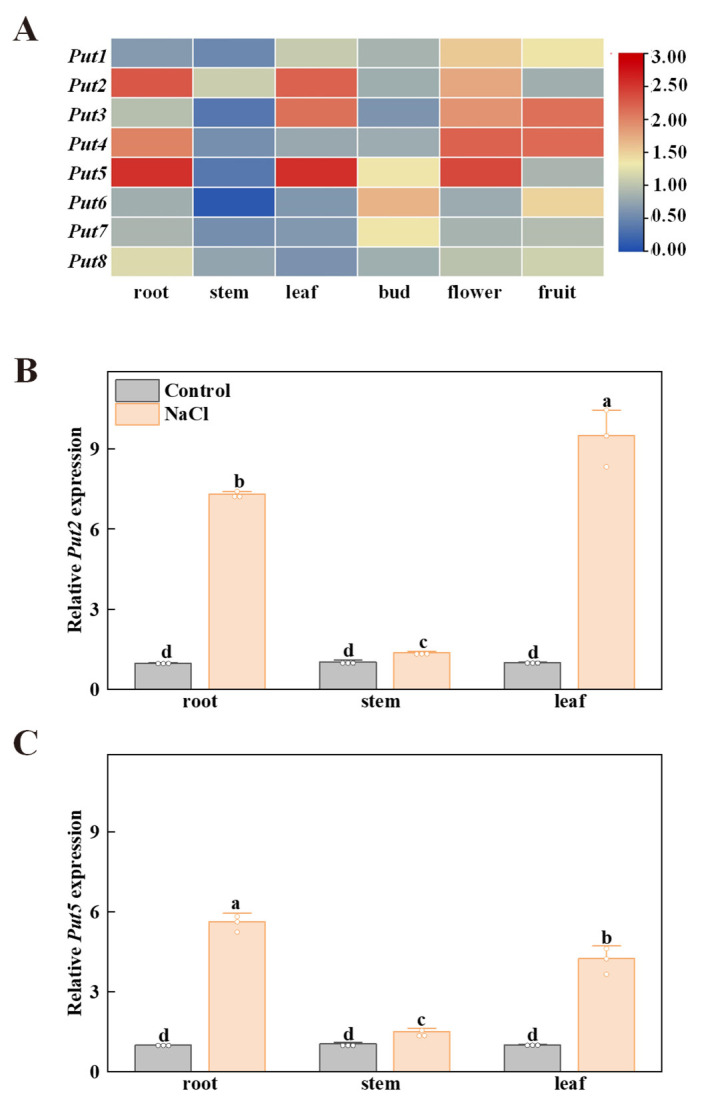
The expression profiles of the tomato Put family of genes in different tissues. (**A**) Heatmap representation of the relative expression of *Put* genes in different tomato tissues. The scale (0 to 3) represents the expression level (from low to high). (**B**,**C**) The spatial expression of *Put2* and *Put5* were performed by qRT-PCR in different organs of WT plants without NaCl (Control) and salt stress (NaCl) (7 days salt treatment). The WT expression in the control condition was set to 1. The data in B and C are presented as mean values ± SD; n = 3. Different letters indicate significant differences between treatments (*p* < 0.05, Duncan’s multiple range test). Three independent experiments were performed with similar results.

To research the functions of the Put family of genes in the tomato, the expression patterns of *Put* in various tissues (e.g., root, stem, leaf, bud, flower, and fruit) were performed by qRT-PCR. As shown in Figure 3, the transcripts of *Put2* and *Put5* had high levels in the leaves and roots, *Put3* exhibited high levels of expression in the leaves and flowers, while *Put4* showed a high level of transcript expression in the roots and flowers. In addition, all *Put*, except *Put6*, *Put7* and *Put8*, were highly expressed in flowers. Subsequently, the spatial expression pattern of *Put2* and *Put5* was analyzed by qRT-PCR in vegetative tissues of WT plants with salt stress. *Put2* and *Put5* showed increased expression in organs of plants under salt stress, with the highest level of *Put2* transcripts in the leaves, and the highest level of *Put5* transcripts in the roots. After 7 days of salt treatment, the *Put2* gene is significantly more highly induced than *Put5* in the leaves (Figure 3B,C).

### 3.6. Functional Analysis of Put Genes in Yeast

**Figure 4:**
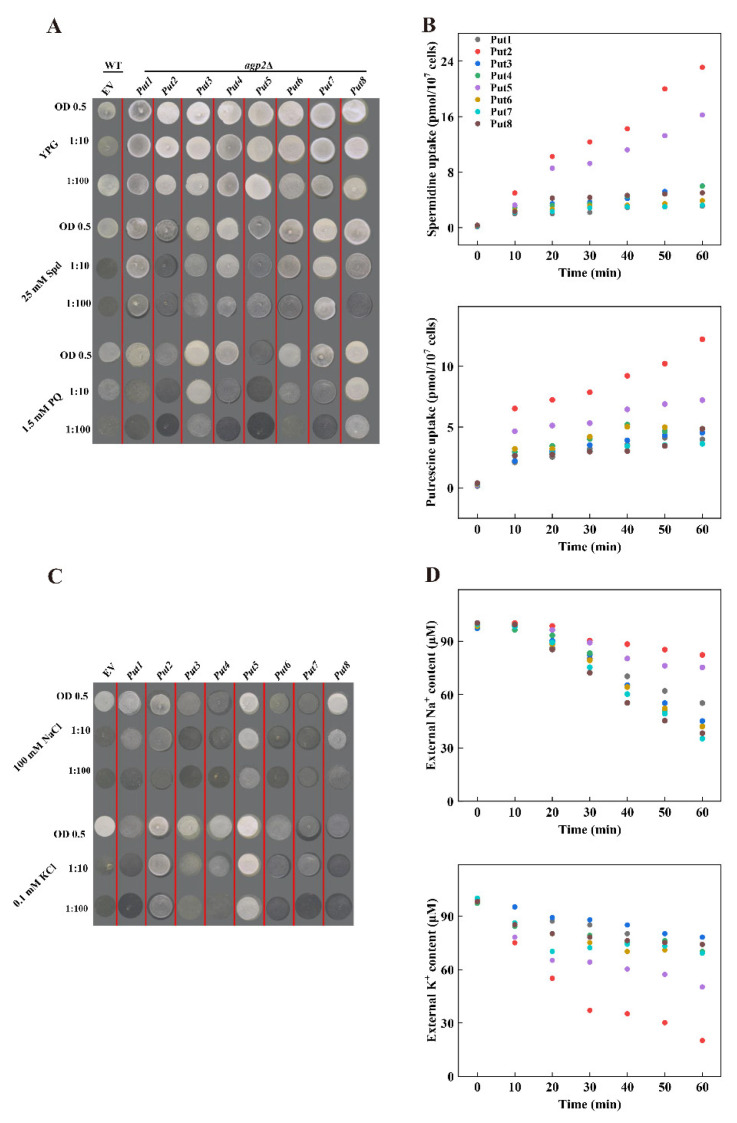
Comparison of polyamines, Na^+^, and K^+^ uptake among tomato *Puts* in yeast. (**A**) Functional complementation of tomato *Puts* in the yeast mutant *agp2*Δ. *agp2*Δ-*Puts* vector strains were grown overnight on SC medium supplemented with 2% galactose. Cell suspensions (the starting OD_600_ is 0.5) were serially diluted as indicated and 3 μL of each were spotted onto YP-galactose plates containing 25 mM spermidine or 1.5 mM paraquat. Plates were photographed after 3–4 days of incubation at 30 °C. The data are representative of one of three independent experiments. EV, empty vector. (**B**) Time course uptake of Spermidine and Putrescine for 0, 10, 20, 30, 40, 50, and 60 min to determine the intracellular amount of Spermidine or Putrescine. (**C**) Functional analysis of tomato *Puts* in the yeast mutant G19 (Δ*ena1–4*) (does not mediate Na^+^ uptake) under NaCl treatment, and in the yeast mutant CY162 (a K^+^ uptake-deficient mutant strain) under deficiency medium. Cell suspensions (the starting OD600 is 0.5) were serially diluted as indicated and 3 μL of each were spotted onto YP-galactose plates containing 100 mM NaCl or 1.5 mM KCl. Plates were photographed after 3–4 days of incubation at 30 °C. The data are representative of one of three independent experiments. EV, empty vector. (**D**) Time course uptake of Na^+^ and K^+^ for 0, 10, 20, 30, 40, 50, and 60 min to determine the external levels of Na^+^ or K^+^.

To examine the potential function of these proteins in polyamine transport, cDNA fragments containing the ORF were cloned and introduced into the yeast expression vector pYES2, driving expression under the *GAL1* promoter. High concentrations of polyamines or paraquat are toxic to wild-type yeast strains, whereas mutant *agp2Δ* lacking the polyamine uptake transporter protein impairs the sensitivity to the high concentration of polyamines. The candidate positive polyamine transporter was introduced to the yeast mutant *agp2*Δ. The expression of polyamine transporter proteins (Put1, Put3, Put6, and Put7) did not affect the phenotype of the *agp2*Δ mutant, however, among the eight Put proteins, Put2 and Put5 transformants were more sensitive than other Put transformants under 25 mM Spermidine conditions. The expression of Put2 or Put5 in the *agp2*Δ mutant conferred sensitivity to 25 mM Spermidine (Figure 4A). In addition, transformants appeared sensitive to the polyamine’s analog PQ, showing that these proteins are also involved in the uptake of PQ.

Further analysis of these genes in light of their competence to transport polyamine was done by incubating with the liquid AP medium supplemented with either 15 μm Spermidine or Putrescine. Transformants of these *Put* genes improved the ability to transport Spermidine or Putrescine than in those of the *agp2*Δ mutant. These transformants possessed higher uptake of Spermidine or Putrescine relative to that mediated by the *agp2*Δ mutant. The Put2- and Put5-transformants were effective for Spermidine and Putrescine, respectively. The uptake ability was more pronounced for Put2 than for Put5 (Figure 4B).

The yeast mutant G19 (Δ*ena1–4*) (does not mediate Na^+^ uptake) and CY162 (K^+^ deficient strain) strain were used to further disclose whether Put could transport Na^+^ or K^+^. The growth status had a significant difference between G19 transformed with Put2 or Put5 than the G19 empty, Put5 had a better growth status, whilst other Put trans-yeast strains were not significant different to the G19-empty vector under 100 mM NaCl treatment (Figure 4C). Similarly, under 0.1 mM KCl, CY162 yeast transformed with Put2 or Put5 had a better-growing status compared to the CY162-empty vector, while other Put trans-yeast strains grew similarly to the CY162-empty vector, indicating that Put2 could transport K^+^, and Put5 could transport Na^+^ and K^+^ (Figure 4C). Furthermore, the ion depletion assay exhibited that K^+^ ions decreased significantly faster than Na^+^ inoculated into the liquid AP medium with 200 μM NaCl + 200 μM KCl, confirming the role of Put2 in Na^+^ and K^+^ transport (Figure 4D).

### 3.7. Put2 Is a Positive Regulator Protein of Tomato Plant Salt Tolerance

**Figure 5:**
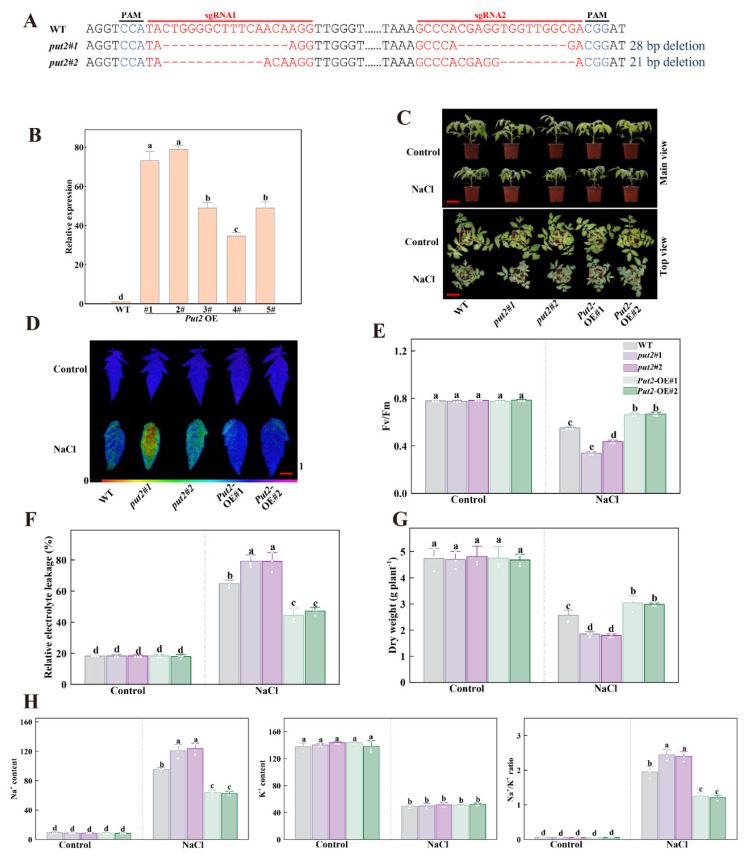
Put2 positively regulates salt tolerance. (**A**) Genotyping of mutations in *put2#1* and *put2#2*. Red letters indicate the target sites, ‘-’ represent sequence deletion, and blue letters represent the protospacer adjacent motif (PAM). (**B**) qRT-PCR analysis of *Put2* transcript levels in WT and Put2 OE (1#, 2#, 3#, 4# and 5#). (**C**) Phenotypes of WT, *put2* mutants, or *Put2* OE lines after exposure with or without salt stress for 7 days. (**D**) *Fv/Fm* in WT, *put2* mutants, or *Put2* OE lines leaves after exposure with or without salt stress for 7 days. The false-color code depicted at the bottom of image range from 0 (black) to 1.0 (purple), showing the level of damage in the leaves. (**E**) Quantitative analysis of *Fv/Fm* as shown in (**D**). (**F**) The relative electrolyte leakage and (**G**) dry weight in WT, *put2* mutants, or *Put2* OE lines after exposure with or without salt stress for 7 days. (**H**) Ion content and Na^+^/K^+^ ratio in shoots of WT, *put2* mutants, or *Put2* OE lines after exposure with or without salt stress for 7 days. Data are presented as mean values ± SD; n = 3. Different letters indicate significant differences between treatments (*p* < 0.05, Duncan’s multiple range test). At least three independent experiments were performed.

We focused our present study on Put2, since *Put2* was highly expressed in leaves after 7 days of salt stress. Furthermore, Put2 had the highest uptake ability of polyamines among the eight Puts and overexpression of *Put2* increased salt tolerance in yeast (Figure 3 and Figure 4). To further determine the role of Put2 in salt stress, as shown in Figure 5, we generated five overexpression lines (named OE#1 to OE#5) and selected two lines (OE#1 and OE#2) for further study after examining Put2 mRNA levels. Meanwhile, two lines of *Put2* mutants (*put2*#1 *and put2*#2) were generated by CRISPR/Cas9 technology, which induced frameshift mutations. Next, we compared the salt tolerance of these tomato plants. In comparison to WT plants, the *Put2*-OE#1 and *Put2*-OE#2 plants exhibited decreased sensitivity to salt stress with less wilted leaves, higher *Fv/Fm* and dry weight, and lower REL after a salt treatment for 7 days. Whereas *put2* mutants displayed salt hypersensitivity with lower *Fv/Fm* and higher REL than WT. These results suggest that Put2 positively regulates salinity tolerance.

To explore whether Put2 responds to salinity stress through mediating the Na^+^ balance, we further analyzed the Na^+^ and K^+^ contents in WT, *put2* mutants, and *Put2-*OE plants subjected to NaCl treatment. K^+^ and Na^+^ content did not differ significantly among the genotypes examined under normal conditions. However, after treatment with 200 mM NaCl for 7 days, in comparison to WT plants, the levels of Na^+^ in shoots parts of the *put2* mutants were much higher, the K^+^ content was significantly lower in mutants, and so the ratios of Na^+^/K^+^ were increased. Conversely, the contents of Na^+^ and the Na^+^/K^+^ ratio in the *Put2*-OE plants were lower, and the levels of K^+^ were higher (Figure 5H).

The transcription levels of encoding salt response genes such as *SOS1-3* and *NHX1-3* were examined in the WT, *put2* mutants, and *Put2*-OE plants using qRT-PCR. The mRNA abundance of *SOS1-3* and *NHX1-3* in the mutants was lower than that in WT. On the contrary, the transcript levels of these genes were significantly increased in *Put2*-OE plants. These results demonstrated that Put2, as a positive regulator, is mediated by Na^+^/K^+^ homeostasis (Figure S2).

### 3.8. Put2 Improves Salt Tolerance by Facilitating Polyamines Synthesis

**Figure 6:**
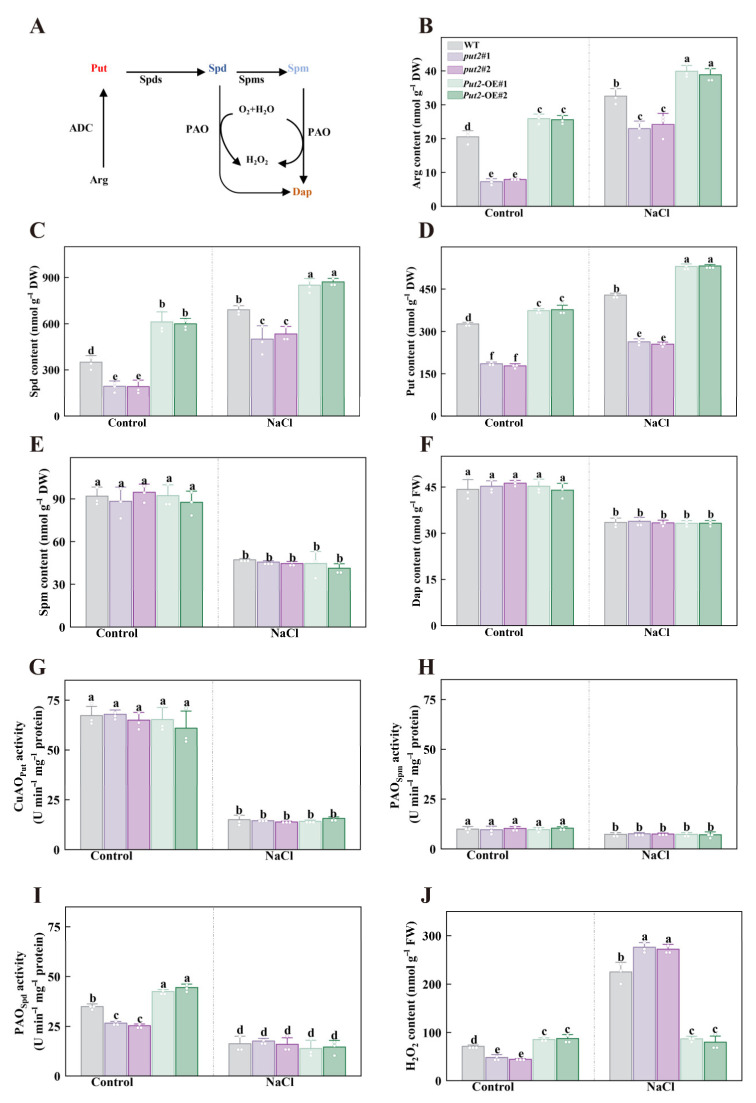
The role of Put2 in the regulation of polyamine metabolism and H_2_O_2_ content. (**A**) Simplified scheme of polyamine biosynthesis relevant to this study in plants; Arg, arginine, ADC, arginine decarboxylase, Put, putrescine, Spds, spermidine synthase, Spd, spermidine, Spms, spermine synthase, Spm, spermine, PAO, polyamine oxidase, Dap, 1,3-diaminopropane. (**B**) The Arg content in WT, *put2* mutants, or *Put2* OE lines with or without salt stress for 7 days. The content of the polyamines Spd (**C**), Put (**D**), Spm (**E**), and Dap (**F**) in WT, *put2* mutants, or *Put2* OE lines with or without salt stress for 7 days. The Put-dependent CuAO (**G**), Spd-dependent PAO (**H**), and Spm-dependent PAO (**I**) enzymatic activity in WT, *put2* mutants, or *Put2* OE lines with or without salt stress for 7 days. (**J**) The H_2_O_2_ content in WT, *put2* mutants, or *Put2* OE lines with or without salt stress for 7 days. Data are presented as mean values ± SD; n = 3. Different letters indicate significant differences between treatments (*p* < 0.05, Duncan’s multiple range test). At least three independent experiments were performed.

Previous studies reported that polyamines uptake proteins can facilitate polyamines synthesis, at least in rosette leaves of *Arabidopsis thaliana* plants (Ahmed et al., 2017), which enlightened us to next investigate the polyamine metabolites. In the polyamine synthesis pathway, key representative molecules (Put, Spd, Spm, and Dap) are derived from the amino acid Arg (Figure 6A). Significantly, there was a considerable increase in the concentrations of Arg (the Put/Spd/Spm precursor), Spd, and Put in the leaves of *Put2*-OE plants, but the content in the *put2* mutants were lower than those of WT plants in the absence of salt stress. When exposed to the salt treatment, the endogenous content of Arg, Spd, and Put increased significantly in *Put2*-OE plants than that of WT and *put2* mutants, whereas the levels of these polyamines in *Put2*-OE plants were still considerably higher than those in the WT plants (Figure 6B–D). Additionally, the Spm and Dap content showed no significant differences among the WT, *put2* mutants, and *Put2*-OE lines (Figure 6E,F).

In leaves, knockout or gain of function of Put2 did not influence the Put-dependent PAO and Spm-dependent PAO activity in the absence or presence of salt treatment (Figure 6G,H). By contrast, *Put2*-OE plants showed an increase in Spd-dependent PAO activity, whereas *put2* mutants exhibited a decrease in this activity, relative to WT plants under normal conditions. During salinity stress, there were no significant changes in the activity of Spd-dependent PAO between WT, *put2* mutants, and *Put2*-OE lines (Figure 6I). Since H_2_O_2_ is one of the PAO reaction products, the levels of H_2_O_2_ were measured. The leaves of *Put2*-OE plants showed an evident increase in H_2_O_2_ content in the absence of salt stress, whereas the largest rise was shown in *put2* mutants in the presence of 200 mM NaCl (Figure 6J), proving that *put2* mutants experience more severe oxidative damage from salt stress than WT and *Put2*-OE plants. Thus, it is possible that Put2-induced H_2_O_2_ production is closely associated with PAOs under normal conditions. On the other hand, Put2 contributes to the decrease of H_2_O_2_ content under salt conditions, which declined oxidative damage and enhanced salt tolerance. These results demonstrate that Put2 is required for some polyamines metabolism intermediates (including Arg, Spd, and Put), as well as the enzyme (PAO).

### 3.9. Put2 Decreases ROS Levels under Salinity Stress

**Figure 7:**
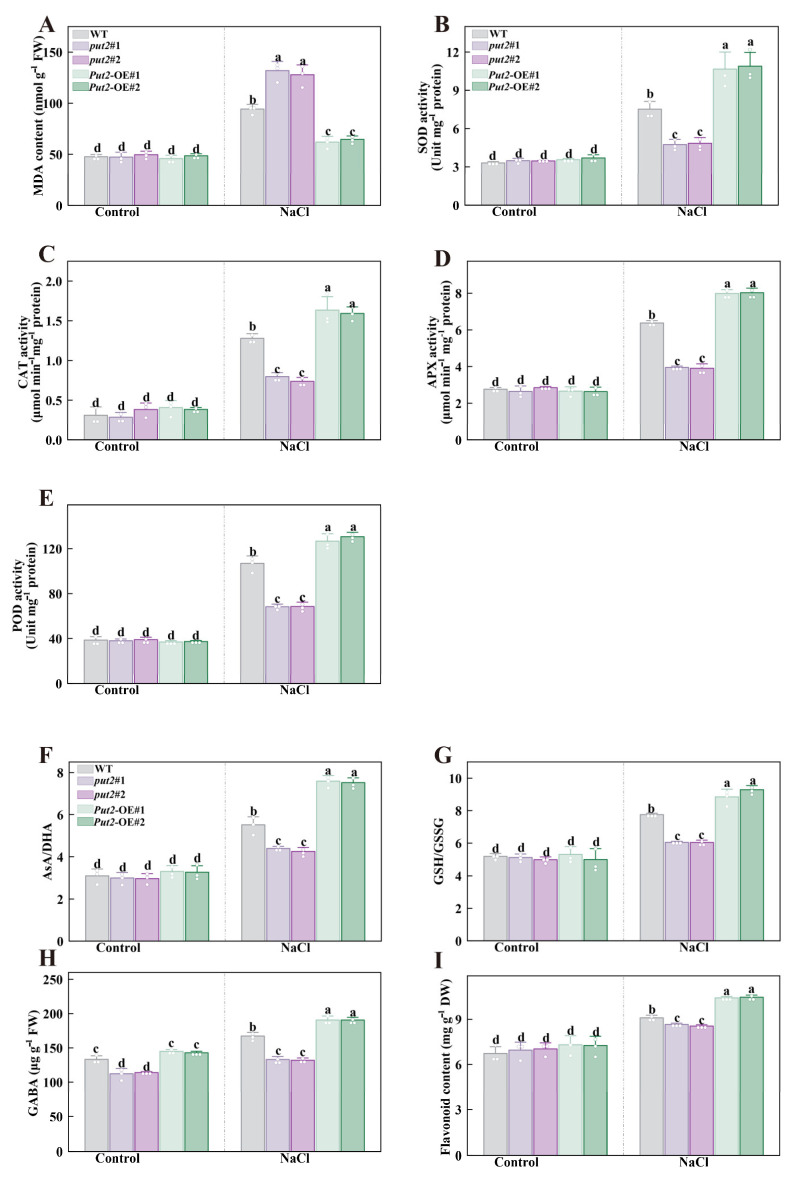
Put2 enhances salt tolerance via positively regulating ROS-scavenging enzyme activity and nonenzymatic antioxidant process. The MDA content (**A**), SOD (**B**), CAT (**C**), APX (**D**), and POD (**E**) activity in WT, *put2* mutants, or *Put2* OE lines with or without salt stress for 7 days. (**F**) The ratio of ascorbic acid (AsA) to dehydroascorbate (DHA), (**G**) the ratio of glutathione (GSH) and glutathione disulfide (GSSG), (**H**) the gamma-aminobutyric acid (GABA), and (**I**) the total flavonoids in WT, *put2* mutants, or *Put2* OE lines with or without salt stress for 7 days. Data are presented as mean values ± SD; n = 3. Different letters indicate significant differences between treatments (*p* < 0.05, Duncan’s multiple range test). At least three independent experiments were performed.

The production of ROS is known to be increased under stress conditions, and H_2_O_2_ is the most stable ROS. To determine whether Put2 reduces ROS accumulation through antioxidant enzyme and non-enzymatic compound regulation under salinity stress, the following relevant indicators were detected. During salt stress, the level of H_2_O_2_ and MDA, two indicators of oxidative damage during salt stress, were significantly lower in *Put2*-OE plants than in WT; increases in H_2_O_2_ and MDA content were detected in *put2* mutants (Figure 6J and Figure 7A). Subsequently, antioxidant enzymes, including SOD, CAT, APX, and POD, are in the midst of the primary enzyme defense against ROS. The activities of SOD, CAT, APX, and POD were more pronounced in *Put2*-OE than in WT. In contrast, *put2* mutants showed lower antioxidant enzyme activity than WT (Figure 7), which is consistent with lower levels of H_2_O_2_ and MDA in *Put2*-OE than in WT and *put2* mutants. These results indicate that Put2 improves salinity tolerance by reducing oxidative damage.

**Figure 8:**
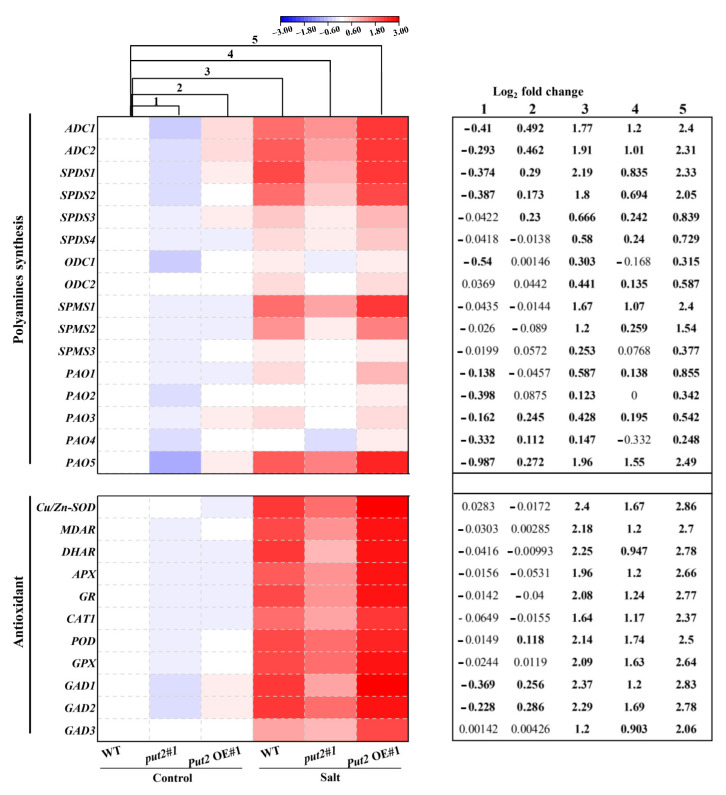
Put2 positively mediates the expression of polyamine synthesis and antioxidant enzyme-encoding genes. Heatmap of polyamine synthesis and antioxidant enzyme-encoding genes. Levels were differentially modified in *put2* vs. WT plants, and *Put2* OE vs. WT plants under normal conditions or salinity stress. Log_2_ fold changes (relative to expression levels in sample of WT plants under control treatment) are shown with a color scale. A chart on the right side of the heatmap shows log_2_ fold change of expression levels in *put2* (mutant) compared to those in WT plants before salt treatment (column 1), log_2_ fold change of expression levels in *Put2* OE (overexpression) compared to those in WT plants before salt treatment (column 2), log_2_ fold change caused by salinity treatment in WT plants (column 3), and log_2_ fold change caused by salinity treatment in *put2* (column 4) or *Put2* OE (column 5). Statistically significant differences are indicated in bold font (*p* < 0.05).

To confirm whether the Put2-mediated ROS decrease is regulated by non-enzyme compounds, we determined the effects of glutathione redox homeostasis, GABA, and flavonoid contents in *put2* mutants and *Put2*-OE plants, which however, revealed no difference from the WT under normal conditions, except for GABA. The GABA content in *put2* mutants was significantly lower than that of WT and *Put2*-OE plants before salt treatment. Overexpression of *Put2* also considerably elevated the AsA/DHA and GSH/GSSG ratios, as well as the content of GABA and flavonoid, while knockout of *put2* compromised the increase in these parameters compared to that in the WT under salinity conditions (Figure 8).

### 3.10. Put2 Triggers Upregulation of Polyamine Synthesis and Is Related to Detoxification Gene Expression

To investigate the transcriptional regulation of polyamine synthesis and detoxification by Put2, we examined the transcript levels of 27 genes involved in polyamine synthesis, ROS detoxification, and GABA synthesis by qRT-PCR in leaves under normal or salinity conditions. qRT-PCR analysis of polyamine synthesis genes in *put2*#1 mutant and *Put2*-OE#1 plants revealed that the set genes of encoding polyamine synthesis (including *ADC1*, *ADC2*, *SPDS1*, *SPDS2*, *SPMS1*, *SPMS2*, *PAO1*, *PAO3,* and *PAO5*) were upregulated in *Put2*-OE#1 plants; in contrast, they were downregulated in *put2*#1 mutants Furthermore, salinity treatment caused relative increases in the expression of polyamine synthesis genes only in WT and *Put2*-OE#1 plants (column 3 vs. column 1, and column 5 vs. column 3), while they decreased in *put2#*1 mutants (column 4 vs. column 3). Collectively, the analysis of differentially expressed genes also suggests that the modifications in gene expression in response to the stress conditions are more intense in the *Put2*-OE#1 plant. Additionally, we noticed an interesting finding with the greatest upregulation of the *PAO5* gene in the five *PAOs* genes in the *Put2*-OE#1 plant under normal conditions, which may be a mechanism for the relatively higher PAO activity and levels of H_2_O_2_ in *Put2* OE plants (Figure 6J and Figure 8).

Similarly, the transcriptional levels of ROS detoxification-related genes, including *Cu/Zn-SOD*, *MDAR*, *DHAR*, *APX*, *GR*, *CAT1*, *POD,* and *GPX*, were further upregulated in the *Put2*-OE#1 plant compared with WT plants after the salinity treatment. By contrast, the induction of ROS detoxification-related genes in response to salt stress was compromised in *put2* mutants; increased ROS-scavenging capacity failed in the *put2*#1 mutant (Figure 8). In addition, the expression of *GAD1*, *GAD2,* and *GAD3*, encoding glutamate decarboxylase, likely the key enzyme for GABA biosynthesis in the tomato, was induced in the *Put2*-OE#1 plant before salt treatment. Accordingly, the transcript levels of *GADs* were further enhanced after salt treatment in the *Put2*-OE#1 plant. Notably, increases in the expression of *GADs* in response to salt stress were suppressed in the *put2*#1 mutant. The enhanced *GADs* transcripts and GABA content was observed in the WT and *Put2*-OE plants but not in the *put2* mutants after salt stress (Figure 7 and Figure 8). Thus, the results indicate that Put2 participates in enhanced salt tolerance by mediating the ROS-scavenging capacity.

## 4. Discussion

Polyamines, which are small polycationic molecular regulators and signaling molecules, not only orchestrate fundamental growth and development in plants, but also induce a series of stress cascades [38]. Polyamines are involved in various pathways in plants and lessen the lethal effects of abiotic stresses by regulating transcription factors, hormonal responses, antioxidant enzymes, and the activation of signaling cascades [39,40]. Polyamine biosynthesis and degradation play important roles in various abiotic stress tolerance pathways and are closely associated with coping with ROS production [41]. The polyamine transporter belongs to the mammalian L-type amino acid transporter family, like polyamine synthesis and metabolism protein, which plays a critical role in plant growth, development, and the stress responses [42]. Though Put has been analyzed in *Arabidopsis*, rice, and the sweet orange, the effects of Put on abiotic stresses in the tomato have not been reported [43,44]. Here, we looked at the physiological and molecular function mechanisms of Put in the resistance to abiotic stress in the tomato. Eight Put proteins in the tomato were identified and characterized at the complete genome level through alignment with *Arabidopsis* Put proteins. Then, extensive analyses on the tomato Put proteins, including phylogenetic development, gene and protein structure, physiochemical characteristics, motifs, miRNAs, and cis elements, and their substrates were performed; and the effects of Puts on abiotic stress tolerance was evaluated in yeast and tomatoes. Furthermore, we demonstrated the importance of the Put2 mediation of polyamine metabolism and antioxidant capacity, and that overexpression of *Put2* increased polyamines to influence the homeostasis of antioxidant capacity, showing that Put2 was a positive regulator for salinity stress in tomatoes. These findings enlarge the comprehension of Put family members, which may be employed in breeding for genetic modification and the development of abiotic stress tolerant crops.

### 4.1. Identification of Tomato Put Family

The tomato Put proteins were divided into four groups, which have a high similarity to those in rice and *Arabidopsis* (Figure 1). Differences among these proteins are probably due to environmental impacts, and analysis of the structure and motif in these genes indicated that *Put* genes disclosed a close exon-intron and motif structure, showing that a closer evolutionary pattern in these genes and diverse functional relationships also exist among the other group members (Figure 1). Interestingly, protein analysis indicated that the PotE (Putrescine-ornithine antiporter) motif is highly conserved, suggesting that it may be involved in amino acid and polyamine transport (Figure 1C) [45,46]. A series of cis elements is a specific sequence at the promoter region of a given gene, which is involved in the expression of protein-coding transcripts and is mediated by transcriptional regulation and small RNAs. Repression and activation of gene expression by binding with these cis elements are a general means of modulating various life processes [47]. Plant miRNAs have a function in regulating related genes that are involved in response to abiotic stresses [48]. miR390 was strongly induced after exposure to salinity during lateral root formation in poplar and positively regulated auxin signaling subjected to salt stress [49]. miR6024 negatively mediates the resistance genes and defense system, facilitates disease by the necrotrophic pathogen *A. solani,* and perturbs immunity in the tomato [50]. The module of miR164a-NAM3 affords cold resistance by increasing ethylene production in the tomato [51]. Tomato Put was targeted by miRNAs including miR390, miR6024, and miR164a, which might be associated with various stress responses (Table 2). We also discovered several types of conserved cis-regulatory elements in the promoter regions of Put; these cis elements are associated with transcriptional regulation of the core gene network, and of plant growth and development [52]. The *Put* genes of the tomato contain various cis elements including stress, hormone, light, auxin, GA, ABA, and methyl jasmonate (MeJA) responsive elements (Figure 1E). The presence of numerous abiotic stress-specific cis-regulatory motifs and hormonal cis elements implicate these genes stimulating the hormone signaling pathways and providing stress tolerance in the tomato [47]. Of note, the cis elements activate their downstream genes after binding to specific transcription factors, playing an important amplifier role during various abiotic/biotic stresses. Meanwhile, we also speculate that Put is involved in salinity stress since these cis-regulatory elements are also closely implicated in salinity tolerance [53].

### 4.2. Expression Profiles of the Put Gene Family after Treatment with Various Hormones, Polyamines, and Abiotic Stresses, in Different Tissues

A gene expression profile can provide critical symbols for its biological functions. Here, we examined the expression pattern of the *Put* genes via qRT-PCR under treatment with exogenous hormones and polyamines, as well as abiotic stress conditions. We observed the *Put* genes appeared to be upregulated in ABA treatment. On the other hand, all Put genes, except for *Put7*, were induced by SA. *Put6*, *Put7,* and *Put8* were inhibited initially after GA_3_ treatment. The expression of five *Put* (*Put4*, *Put5*, *Put6*, *Put7,* and *Put8*) genes were downregulated, and the other *Put* (*Put1*, *Put2* and *Put3*) genes were induced in leaves at 3 and 6 h of ET treatment. Similarly, the critical role of growth factors and hormones in increasing polyamine transport rates in mammalian cells has been demonstrated [54]. Together with the presence of hormone-responsive cis elements and altered transcripts levels after hormone treatment, this implies that Put may have a crucial role in the hormone regulatory pathway. Furthermore, the differential expression profiling of *Put* in response to polyamine treatment was evaluated. All *Put* genes are involved in exogenous polyamines treatment; the *Puts* exhibited varied patterns in response to the same polyamine. For instance, *Put2* and *Put5* were dramatically upregulated, while the other genes were slightly induced after spermidine treatment. However, putrescine resulted in suppression of these *Put* genes. Additionally, spermine led to the upregulation of *Puts*, especially *Put2* and *Put5*. On the other hand, the qRT-PCR analysis revealed pronounced effects of the spermidine and spermine-induced *Put* gene expression (Figure 2B). Since polyamines are used as substrates required for the polyamine uptake proteins [43], the different polyamine responses may involve the substrate specificity of polyamine transport and homeostasis. In fact, rice Put1 is a specific and high-affinity spermidine uptake transporter involved in polyamines uptake, leading to the accumulation of polyamines in yeast [55]. The lower affinity of Put may be the reason for its higher proportion in the free state. Therefore, spermidine, spermine, and putrescine, may severe as Put substrates. Interestingly, after salt and drought stress, *Put2* and *Put5* were significantly induced compared with the others. However, their expression was significantly inhibited under cold and heat temperature stress (Figure 2C). Altogether, these results indicate that *Put* genes are potentially involved in hormone and polyamine induction, as well as in response to salinity stress.

The *Put* genes exhibited a divergent expression pattern in different tissues. *Put2* and *Put5* displayed the highest expression in leaves and roots, and all genes had high expression in flowers and fruit (Figure 3A). Similarly, the Put family appears to have a distinct tissue expression profile in *Arabidopsis* and *Citrus sinensis* [43,44], and a divergent pattern of intracellular localization [13], which implied specialization in a spatial manner. Furthermore, *Put2* was more significantly induced by salt stress than *Put5* in leaves and roots, indicating that the functional role of *Put2* related to salt stress may be important. In the tomato, the functional role of *Put2* in abiotic stress tolerance remains largely unknown.

### 4.3. Put2 Contributes to Polyamins Biosynthesis and Catabolism Associated with Salt Tolerance

Although our results above have shown that Put is involved in abiotic stress, its function is yet to be understood, especially regarding polyamine transport and salt stress. Previous studies have shown that yeast is an excellent heterologous expression system to study the function of genes in polyamine transport and salt stress [43]. Here, we used the yeast model to preliminarily investigate their function in polyamine uptake and salt stress tolerance (Figure 4). The results showed that transformants of *agp2*Δ mutants expressing *Put2* and *Put5* had higher sensitivity to spermidine and paraquat, indicating that both function as an importer. Here, we also showed that the yeast *agp2*Δ cells’ capacity to transport paraquat may be compensated by Put2 and Put5 (Figure 4A), since paraquat is transported by the polyamine transport system [12]. A time course absorption experiment directly provided evidence that *Put2* and *Put5* encoded a transporter that can regulate polyamines import, with high activity of polyamine uptake for Put2 (Figure 4B). Furthermore, overexpression of *Put2* increased salt tolerance in yeast, hampered the influx of Na^+^, and enhanced K^+^ uptake (Figure 4C,D). Indeed, polyamine transporters have recently been linked to the regulation of salt stress through promoting Na^+^ efflux and K^+^ channels [56]. Thus, combining the previous results in this article (Figure 2, Figure 3 and Figure 4), we speculate that the induction of *Put2* expression by salt may be regulating the polyamines and Na^+^/K^+^ homeostasis to alleviate salt damage.

To further validate the function of Put2, the *put2* mutants and overexpression lines were generated (Figure 5A,B). Our results showed that *put2* mutants were more shriveled than WT plants under salinity stress, whereas overexpression elevated salt tolerance (Figure 5C). Likewise, *Put2*-OE plants displayed increased levels of Fv/Fm and dry weight, and reduced levels of relative electrolyte, which agrees with an increase in salt tolerance and a decrease in Na^+^ content and the Na^+^/K^+^ ratio (Figure 5D–H). Similarly, Put3 is critical for Na^+^ and K^+^ homeostasis by physically interacting with SOS1 and SOS2, forming a complex with SOS2 under stress conditions [56]. As such, the induction of *SOS1-3* and *NHX1-3* in *Put2*-OE plants could also synergistically activate the SOS1 and SOS2 (Figure S2). Thus, subsequent increases in the Put2 activity would enhance salt tolerance by activating the Na^+^/H^+^ exchange activity.

Polyamines, an important regulator in the plant kingdom, are necessary for plant growth, development, and the stress response. The dynamic balance of polyamines in the plant is stringently regulated by polyamine synthesis, degradation, and transport [57,58]. The latter was previously involved in subcellular polyamine transport through the complementation experiment in yeast [55], and the transport of paraquat in *Arabidopsis* [12]. While the implication of *Put2* in polyamine biosynthesis and catabolism was not noted. Here, under control conditions, overexpression of *Put2* increases the endogenous Arg, Spd, and Put content, which failed to increase in *put2* mutants. Upon salt stress, meanwhile, the polyamine content in WT plants performed much better than *put2* mutants, and *Put2*-OE plants were better than the WT (Figure 6A–D). NaCl supply treatment also increased the activation of polyamine synthesis-related genes more clearly in *Put2*-OE plants (Figure 8), demonstrating the important function of Put2 in the polyamine biosynthesis process. On the other hand, considering polyamine catabolism, the higher activity of PAO_spd_ confirmed the acceleration of polyamine catabolic reactions in *Put2*-OE plants (Figure 6I), and evidenced that Put2 positively regulates PAO activity. In addition, two main sources of ROS were indicated to exist in plants, including NADPH oxidases and polyamines catabolism by PAO activity [8,59]. In *put2* leaves, H_2_O_2_ was lowered with respect to WT plants under normal conditions; however, an upregulation was observed in *Put2*-OE plants, and likewise PAO activity and *PAO* gene expression were altered (Figure 6J and Figure 8), further demonstrating that Put2 attributes to PAO activity. Accordingly, enhancement of PAO activity has been shown to alleviate salinity damage and increase the polyamines and H_2_O_2_ content [60,61]. Therefore, Put2 may contribute to governing polyamine biosynthesis and catabolism. However, we cannot completely rule out other possibilities, for example, Put2 may be capable of regulating the long-distance and appropriate tissue distribution of polyamines [62].

### 4.4. Put2-Mediated Antioxidant Capacity Establishes Suitable ROS Levels under Salt Stress

PAO activity has been reported to contribute to an increase in salt tolerance through the production of H_2_O_2_ [63]. However, *Put2*-OE plants treated with NaCl showed that the increase in H_2_O_2_ content was lower than in WT plants. Furthermore, the H_2_O_2_ content increased considerably in *put2* mutants accompanied by serious salt stress injury, which correlated inversely with the activities of PAO (Figure 6J). Thus, PAO-induced H_2_O_2_ production was stunted under salt conditions in *Put2*-OE plants; therefore, another mechanism to eliminate H_2_O_2_ must exist. Indeed, plants exposed to salt stress generate a super-excess of ROS, which is highly toxic and can overwhelm the PAO-induced H_2_O_2_, ultimately damaging cellular activity and leading to plant death [8]. In the current study, *Put2*-OE plants exhibited obviously increased activities of antioxidant enzymes after salt treatment. Additionally, the ASA-GSH cycle was also activated by overexpression of *Put2*, which could be another exploration for Put2 enhanced salt tolerance, as the enzymatic system and scavenging procedure could be activated in *Put2*-OE plants (Figure 7 and Figure 8). These findings indicate that Put2 functions as an important regulator linking polyamines and ROS, and affects both the production and elimination of ROS. The results coincided with a previous study showing that Put2 promoted phyA-mediated germination by sensing seed oxidation and protecting the decaying seed from oxidative damage [64]. Therefore, there is strong evidence that Put2 increased salt tolerance probably by promoting antioxidants in plants. A similar report has been conducted showing that CsPUT4 can protect against cold stress by modulating polyamine homeostasis and turning on the antioxidant enzyme defense system in the sweet orange [44]. Meanwhile, GABA and flavonoids, as free radical scavengers, have been exhibited to alleviate salinity damage and heat damage by inducing polyamine enhancement [65,66], which are increased in *Put2*-OE plants under salinity stress; this further demonstrates positive feedback regulation by Put2.

## 5. Conclusions

In this work, eight Put family proteins were found in the tomato, and their chromosomal location, structure, phylogenetic tree, and physiochemical properties were investigated. Furthermore, molecular characterization was performed in yeast to understand their involvement in polyamine uptake and salt stress tolerance. Additionally, the cis elements in the promoter, miRNAs targeting Put, and the expression profiles of *Put* genes in different tissues and their responses to exogenous hormones and polyamines, as well as abiotic stress, were analyzed, proving they may play a vital function in abiotic stress, growth, and development. Furthermore, we show the role of Put2, which, to our knowledge, is the first polyamine uptake protein characterized in the tomato shown to play a role in salinity tolerance. Firstly, in yeast, Put2 was highly tolerant to salt stress, as indicated by less Na^+^ invasion and K^+^ efflux, which also could be attributable to an enhancement in the absorption of polyamines. Importantly, overexpression of *Put2* in the tomato decreased salinity sensitivity, evidenced by enhanced polyamine biosynthesis and catabolism and maintained Na^+^/K^+^ homeostasis, in addition to activated ROS-scavenging enzyme activities and nonenzymatic antioxidant process. These findings shed light on Put2-regulated salinity tolerance in the tomato. Here, we provide comprehensive deciphering of the mechanisms of Put2 for enhancing salt tolerance and some valuable evidence for interpreting the potential functions of tomato Put genes in abiotic stress tolerance.

Clearly, further studies are required to understand the precise function of Put and the upstream and downstream targets of the individual Puts. Generating loss- and gain- of-function mutations and characterizing their roles will provide useful tools, generating new evidence and new findings.

## Figures and Tables

**Table 1:** Put gene identification and characterization in the tomato.

Gene	Gene Code	Chr Number	Location on CHR	CDS_Length	AA_Length	PI	MW(KDa)
Put6	Solyc01g005920	chr1	611660-613377	1077	359	7.96	40.28
Put4	Solyc01g034080	chr1	33621254-33622666	1413	471	6.05	52.67
Put7	Solyc01g111800	chr1	90326196-90327818	1386	462	8.67	51.33
Put2	Solyc08g005540	chr8	411003-415969	1593	531	6.28	58.25
Put3	Solyc08g075710	chr8	57951470-57958170	1605	535	5.41	58.8
Put1	Solyc08g078100	chr8	60059627-60061033	1407	469	8.83	51.71
Put8	Solyc09g092420	chr9	67625291-67628225	1092	364	9.37	41.19
Put5	Solyc10g049640	chr10	45328007-45330412	1473	491	9.28	54.5

**Table 2:** Prediction of miRNAs targeting the Put genes in tomato.

miRNA	Target	Expectation	Target_Site	miRNA_Aligned_Fragment	Alignment	Target_Aligned_Fragment	Inhibition
sly-miR1917	Solyc01g005920	4	400–420	AUUAAUAAAGAGUGCUAAAGU	::::::::::.::: ::.:	CCUUUAGCACUUUUUUCUAGU	Cleavage
sly-miR9479-3p	Solyc01g034080	3.5	180–201	GAGAAUGGUAGAGGGUCGGACC	: :: ::..:.:.:::::::.	UGGCCCACUUUUUGCCAUUCUU	Cleavage
sly-miR6024	Solyc01g034080	4	721–742	UUUUAGCAAGAGUUGUUUUACC	::.: ::::.:: :::::.	CCGAAGAGAACUUUUCCUAAAG	Cleavage
sly-miR171c	Solyc01g034080	4.5	775–795	UAUUGGUGCGGUUCAAUGAGA	:.::: ::.: :::.:::.	GGUUAUUUAAUCCCACUAAUG	Cleavage
sly-miR171a	Solyc01g034080	5	964–984	UGAUUGAGCCGUGCCAAUAUC	: : ::.:.:::: :::.:	CUUUUAGGUAUGGCUGAAUUA	Cleavage
sly-miR9472-5p	Solyc01g034080	5	1262–1282	UUUCAGUAGACGUUGUGAAUA	.::.:.::.: :::.:: ::	CGUUUAUAAUGGCUAUUGCAA	Translation
sly-miR9478-3p	Solyc01g034080	5	1125–1145	UUCGAUGACAUAUUUGAGCCU	:.:.:..:::::. :.:.:	UAGUUUAGGUAUGUUUUUGGA	Cleavage
sly-miR156a	Solyc01g111800	4.5	1089–1109	UUGACAGAAGAUAGAGAGCAC	:.::::: ::.::::::	GGAAUUUCUAUGUUUUGUCAA	Translation
sly-miR156b	Solyc01g111800	4.5	1089–1109	UUGACAGAAGAUAGAGAGCAC	:.::::: ::.::::::	GGAAUUUCUAUGUUUUGUCAA	Translation
sly-miR156c	Solyc01g111800	4.5	1089–1109	UUGACAGAAGAUAGAGAGCAC	:.::::: ::.::::::	GGAAUUUCUAUGUUUUGUCAA	Translation
sly-miR390a-5p	Solyc01g111800	5	1017–1037	AAGCUCAGGAGGGAUAGCACC	: ::::.::.. ::: ::.::	GCUGCUGUCUUGCCUUAGUUU	Translation
sly-miR396a-3p	Solyc01g111800	5	682–702	GUUCAAUAAAGCUGUGGGAAG	::::.: .:::::::: ::	AUUCCUAAGGCUUUAUUCUAC	Cleavage
sly-miR6023	Solyc01g111800	5	599–620	UUCCAUGAAAGAGUUUUUGGAU	:::.::: :: ::::. :::::	AUCUAAAUACACUUUUUUGGAA	Cleavage
sly-miR6024	Solyc08g005540	5	880–901	UUUUAGCAAGAGUUGUUUUACC	::.: :::::: :::::.	CCGAAGAAAACUCUGCCUAAAG	Cleavage
sly-miR6026	Solyc08g005540	5	1181–1202	UUCUUGGCUAGAGUUGUAUUGC	... ::: ::::::.:..::	AUGGAACACCUCUAGUCGGGAU	Cleavage
sly-miR6023	Solyc08g075710	4	824–845	UUCCAUGAAAGAGUUUUUGGAU	:::..:: :::::::. :::::	AUCUGAAUACUCUUUUCUGGAA	Cleavage
sly-miR1916	Solyc08g075710	5	526–545	AUUUCACUUAGACACCUCAA	::: :: ::.::::.:.:	AUGAAAUGGCUGAGUGGAGU	Cleavage
sly-miR390a-5p	Solyc08g078100	3.5	778–798	AAGCUCAGGAGGGAUAGCACC	:: :::::.::::.::::: :	GGAGCUAUUCCUCUUGAGCAU	Cleavage
sly-miR390b-5p	Solyc08g078100	3.5	778–798	AAGCUCAGGAGGGAUAGCGCC	:: :::::.::::.::::: :	GGAGCUAUUCCUCUUGAGCAU	Cleavage
sly-miR6022	Solyc08g078100	5	518–538	UGGAAGGGAGAAUAUCCAGGA	: ::: :::::.::::...	UACUGUCAAUUCUUCCUUUUG	Cleavage
sly-miR156a	Solyc09g092420	3	945–965	UUGACAGAAGAUAGAGAGCAC	:.::::::::.::::::	GGAAUUUCUAUCUUUUGUCAA	Cleavage
sly-miR156b	Solyc09g092420	3	945–965	UUGACAGAAGAUAGAGAGCAC	:.::::::::.::::::	GGAAUUUCUAUCUUUUGUCAA	Cleavage
sly-miR156c	Solyc09g092420	3	945–965	UUGACAGAAGAUAGAGAGCAC	:.::::::::.::::::	GGAAUUUCUAUCUUUUGUCAA	Cleavage
sly-miR319b	Solyc09g092420	5	1013–1033	UUGGACUGAAGGGAGCUCCCU	::: :. :::::::.::.	UAGGAAGUUACUUCAGUUCAG	Cleavage
sly-miR319c-3p	Solyc09g092420	5	1013–1033	UUGGACUGAAGGGAGCUCCUU	:::: :. :::::::.::.	UAGGAAGUUACUUCAGUUCAG	Cleavage
sly-miR396a-3p	Solyc09g092420	5	538–558	GUUCAAUAAAGCUGUGGGAAG	::::.: .:::::::: ::	AUUCCUAAGGCUUUAUUCUAC	Cleavage
sly-miR6023	Solyc09g092420	5	455–476	UUCCAUGAAAGAGUUUUUGGAU	:::.::: :: ::::. :::::	AUCUAAAUACACUUUUUUGGAA	Cleavage
sly-miR164a-5p	Solyc10g049640	5	1195–1215	UGGAGAAGCAGGGCACGUGCA	::.:: .:. ::::::..:	GUCAUGUAUCUGGCUUCUUUA	Translation
sly-miR164b-5p	Solyc10g049640	5	1195–1215	UGGAGAAGCAGGGCACGUGCA	::.:: .:. ::::::..:	GUCAUGUAUCUGGCUUCUUUA	Translation

## Data Availability

Data are contained within the article and Supplementary Material.

## Supplementary Materials

The following supporting information can be downloaded at: https://www.mdpi.com/article/10.3390/antiox12020228/s1, Figure S1. Cartoon of the predicted topology of tomato Put proteins. Transmembrane domains are indicated as blue rectangle. Figure S2. Relative transcript levels of SOS1, SOS2 and SOS3 (A), NHX1, NHX2 and NHX3 (B) gene expression in WT, put2 mutants, or Put2 OE lines after exposure with or without salt stress for 7 days. Data are presented as mean values ± SD; n = 3. Different letters indicate significant differences between treatments (*p* < 0.05, Duncan’s multiple range test). At least three independent experiments were performed. Figure S3. The MDA content in WT, put2 mutants, or Put2 OE lines after exposure with or without salt stress for 7 days. Data are presented as mean values ± SD; n = 3. Different letters indicate significant differences between treatments (*p* < 0.05, Duncan’s multiple range test). At least three independent experiments were performed. Supplemental data S1. The protein sequences of Put in Arabidopsis, rice and tomato, respectively. Supplemental data S2. The Put genes Cis-element distribution. Supplemental data S3. Primers used in this study.
