# Supplementary material for: Genome-Wide Identification and Characterization of the Polyamine Uptake Transporter (Put) Gene Family in Tomatoes and the Role of Put2 in Response to Salt Stress
Source: Antioxidants (Basel). 2023 Jan 18;12(2):228. doi: 10.3390/antiox12020228 (PMC9952195; doi:10.3390/antiox12020228)
Supplement: Supplementary file 1 [file antioxidants-12-00228-s001.zip › antioxidants-2135816-supplementary/Figure S .pdf]

Genome-wide identification and characterization of polyamine uptake transporter (Put) gene family in tomato and the role of Put2 in response to salt stress

Supplemental data

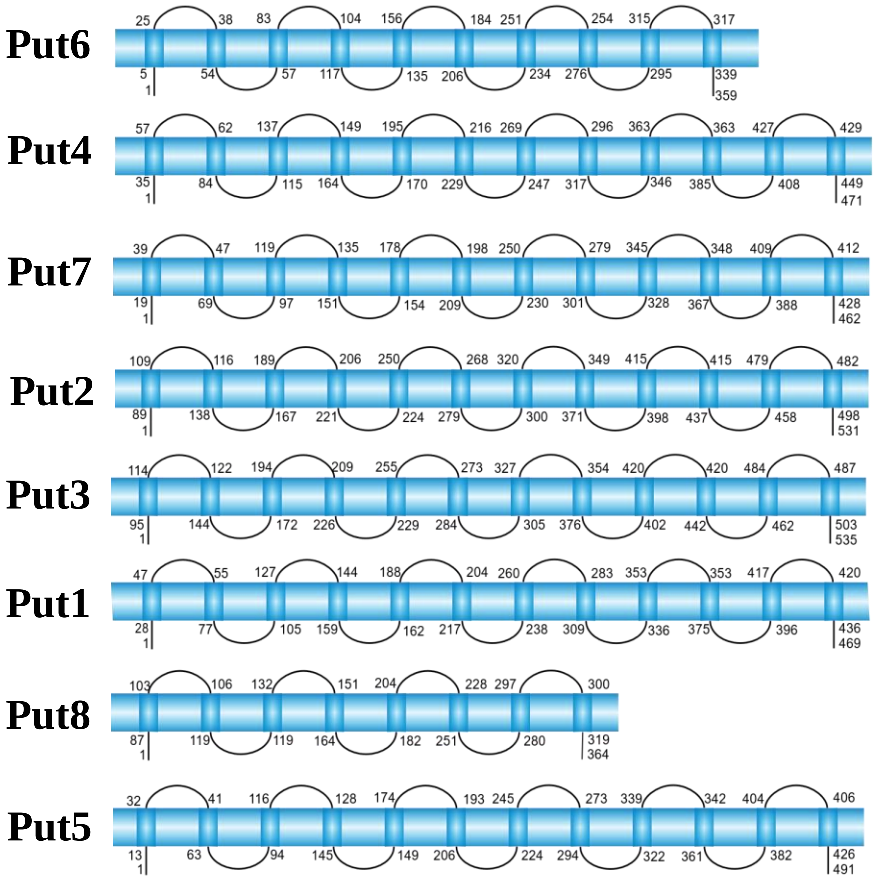

Figure S1. Cartoon of the predicted topology of tomato Put proteins. Transmembrane domains are indicated as blue rectangle.

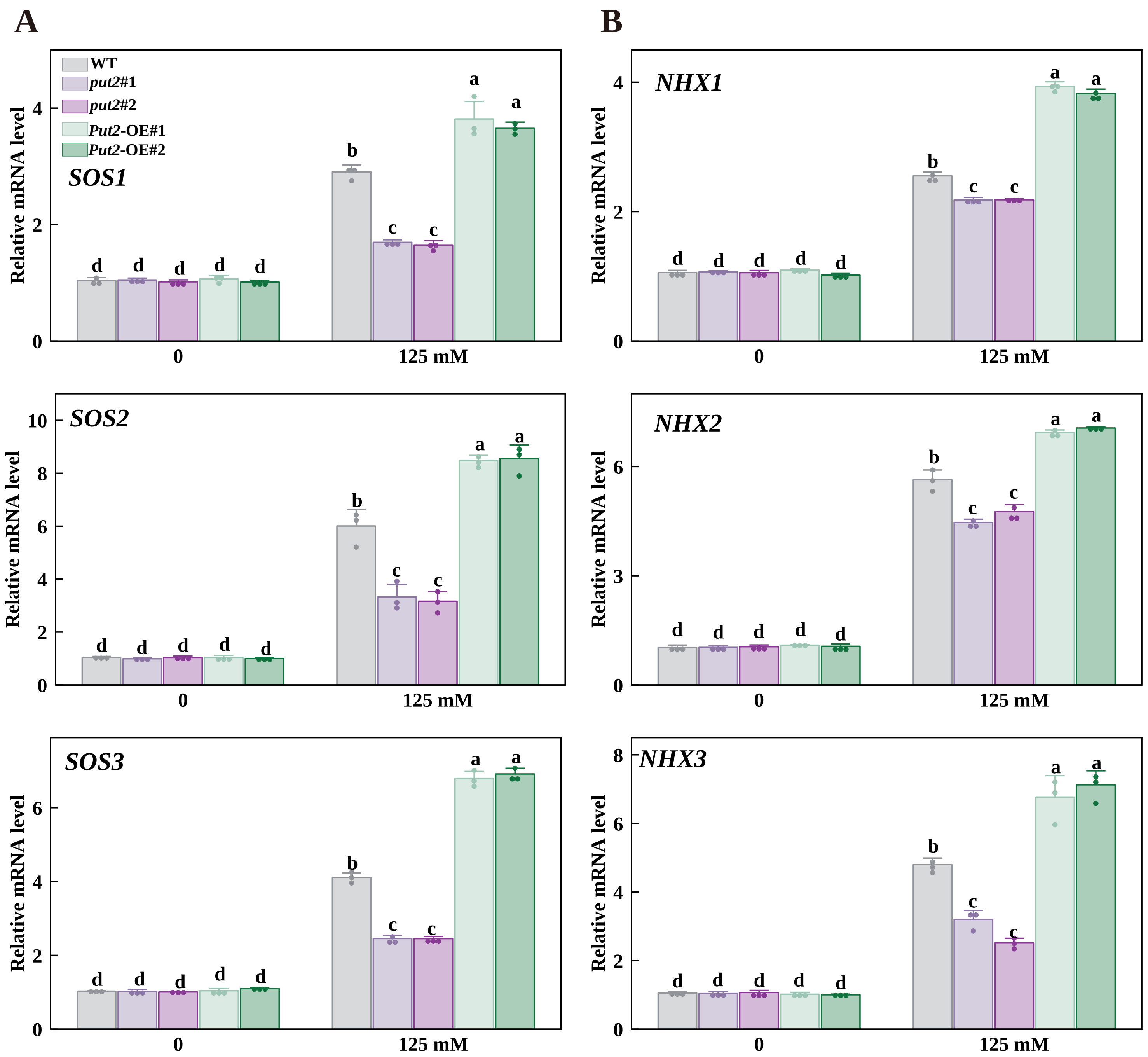

Figure S2. Relative transcript levels of *SOS1*, *SOS2* and *SOS3* **(A)**, *NHX1*, *NHX2* and *NHX3* **(B)** gene expression in WT, *put2* mutants, or *Put2* OE lines after exposure with or without salt stress for 7 days. Data are presented as mean values±SD; n=3. Different letters indicate significant differences between treatments ($P<0.05$, Duncan's multiple range test). At least three independent experiments were performed.

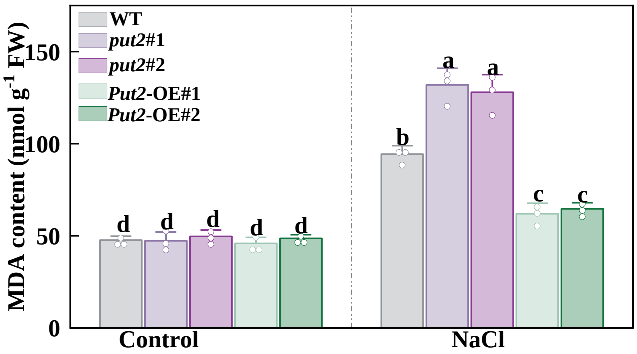

Figure S3. The MDA content in WT, *put2* mutants, or *Put2* OE lines after exposure with or without salt stress for 7 days. Data are presented as mean values±SD; n=3. Different letters indicate significant differences between treatments ($P<0.05$, Duncan's multiple range test). At least three independent experiments were performed.
