# Supplementary material for: Genome-Wide Identification and Characterization of the Polyamine Uptake Transporter (Put) Gene Family in Tomatoes and the Role of Put2 in Response to Salt Stress
Source: Antioxidants (Basel). 2023 Jan 18;12(2):228. doi: 10.3390/antiox12020228 (PMC9952195; doi:10.3390/antiox12020228)
Supplement: Supplementary file 1 [file antioxidants-12-00228-s001.zip › antioxidants-2135816-supplementary/Supplemental data S1.pdf]

Supplemental data S1. The protein sequences of Put in arabidopsis, rice and tomato, respectively.

>At1G31820(AtPut1)
MGDYNMNEFAYGNLYDDDDGDVGGSSKEGNNSIQKVSMLPLVFLIFYEVSGGPFGAEGS
VNAAGPLLALLGFVIFPFIWCIPEALITAEMSTMFPINGGFVVWVSSALGTFWGFQVGWM
KWLCGVIDNALYPVLFLDYLKSAVPALATGLPRVASILILTLLLTYLNYRGLTIVGWTAVFM
GVFSMLPFAVMSLVSIPQLEPSRWLVMDLGNVNWNLYLNTLLWNLNYWDSVSTLAGEVA
NPKKTLPKALCYGVIFVALSNFLPLLSGTGAIPLDRELWTDGYLAEVAKAIGGGWLQLWV
QAAAATSNMGMFLAEMSSDSFQLLGMAELGILPEIFAQRSRYGTPLLGILFSASGVLLLSG
LSFQEIIAAENLLYCGGMILEFIAFVRLRKKHPAASRPYKIPVGTVGSILICVPPIVLICLVIVL
STIKVALVSFVMVVIGFLMKPCLNHMDGKKWVKFSVCSDLAEFQKENLDCEESLLR
>At1G31830(AtPut2)
MQKRRIITVNPSASIEMSQYENNEVPYSSVGADEVPSSPPKATDKIRKVSMLPLVFLIFYEV
SGGPFGVEDSVNAAGPLLALLGFVIFPFIWSIPEALITAEMGTMYPENGGYVVWVSSALGP
FWGFQQGWMKWLSGVIDNALYPVLFLDYLKSGVPALGSGLPRVASILVLTILLTYLNYRGL
TIVGWVAVLMGVFSILPFAVMGLISIPQLEPSRWLVMDLGNVNWNLYLNTLFWNLNYWDS
ISTLAGEVENPNHTLPKALFYGVILVACSYIFPLLAGIGAIPLEREKWTDGYFSDVAKALGG
AWLRWWVQAAAATSNMGMFIAEMSSDSFQLLGMAERGMLPEFFAKRSRYGTPLLGILFS
ASGVVLLSWLSFQEIVAAENLLYCVGMILEFIAFVRMRMKHPAASRPYKIPIGTTGSILMCI
PPTILICAVVALSSLKVAAVSIVMMIIGFLIHPLLNHMDRKRWVKFSISSDLPDLQQQTREYE
ETLIR
>At5G05630(AtPut3)
MTELSSPNLDSASQKPRISTENPPPPPPHISIGVTTGDPATSPARTVNQIKKITVLPLVFLIFYE
VSGGPFGIEDSVKAAGPLLAIVGFIVFPFIWSIPEALITAEMGTMFPENGGYVVWVTLAMG
PYWGFQQGWVKWLSGVIDNALYPILFLDYLKSGIPILGSGIPRVAAILVLTVALTYLNYRGL
SIVGVAAVLLGVFSILPFVVMSFMSIPKLKPSRWLVVSKKMKGVNWSLYLNTLFWNLNY
WDSVSTLTGEVENPSKTLPRALFYALLLVVFSYIFPVLTGTGAIALDQKLWTDGYFADIGK
VIGGVWLGWWIQAAAATSNMGMFLAEMSSDSFQLLGMAERGMLPEVFAKRSRYRTPWV
GILFSASGVIILSWLSFQEIVAAENLLYCFGMVLEFITFVRLRMKYPAASRPFKIPVGVLGSV
LMCIPPTVLIGVIMAFTNLKVALVSLAAIVIGLVLQPCLKQVEKKGWLKFSTSSHLPNLME
>At3G13620(AtPut4)
MAISEASKSSHELPVTTAESSGKKATAKKLTLIPLVFLIYFEVAGGPFGEEPAVQAAGPLLAI
LGFLIFPFIWSIPEALITAELSTAFPGNGGFVIWAHRAFGSFVGSMMGSLKFLSGVINVASFP
VLCVTYLDKLFPVLESGWPRNVCIFASTVVLSFLNYTGLAIVGYAAVVLGLVSLSPFLVMS
AMAIPKIKPHRWGSLGTKKKDWNLYFNTLFWNLNFWDNVSTLAGEVDEPQKTFPLALLI
AVIFTCVAYLIPLFAVTGAVSVDQSRWENGFHAEAAEMIAGKWLKIWIEIGAVLSSIGLFEA
QLSSSAYQLEGMAELGFLPKFFGVRSKWFNTPWVGILISALMSLGLSYMNFTDIISSANFL
YTLGMFLEFASFIWLRRKLPQLKRPYRVPLKIPGLVVMCLIPSAFLVLILVFATKIVYLICGV
MTIGAIGWYFLINYFRKTKIFEFNEVIDDLDNNVNGEHPKVDDHNS
>At3G19553(AtPut5)
MGEEETIVNDENSSKPKPSPKLTLLPLVFLIFYEVSGGPFGVEDSVKSGGGPLLALLGFLIFP
LIWSIPEALVTAELATSFPENGGYVVWISSAFGPFWGFQEGFWKWFSGVMDNALYPVLFL
DYLKHSFPVLDHVAARVPALLVITFSLTYLNYRGLHIVGFSAVVLAVFSLCPFVVMALLAVP

NIRPKRWLFVDTQKINWRGYFNTMFWNLNYWDKASTLAGEVDRPGKTFPKALFGAVLL
VMGSYLIPLMAGTGALSSSTSGEWSDGYFAEVGMLIGGVWLKGWIQAAAAMSNLGLFE
AEMSSDAFQLLGMSEIGMLPAFFAQRSKYGTPTISILCSATGVIFLSWMSFQEIIEFLNFLYA
LGMLLEFAAFVKLRIKKPDLHRPYRVPLNTFGVSMLCLPPSLLVILVMVLAAPKTFLISGVI
IVLGFCLYPFLTLVKEKQWARFIPEETRPVSGVSSESQLDEEHGDESAASLLP

>Solyc08g078100(SlPut1)
MGEFDDGEYAGINEVTSPRENNARKVSVLPLLFLIFYEVSGGPFGVEDTVRAAGPLLALL
GFLVFPFIWSVPEALITAEMGTMFPENGGYVVWVSSALGPYWGFQLGWMKWLSGVIDN
ALYPVLFLDYLKSAIPALGGGLPRVLAVLVLTVVLTYMNYRGLTIVGWVAVSLGILSILPFV
VMGLISIPKLRPSRWLVVDVQSVDWNLYLNTLFWNLNYWDSISTLAGEVHNPKKTLPKA
LFYAVILVVLSYFFPLLIGTGAIPLEHDLWTDGYFSDIAKILGGVWLRVWIQGAAAASNMG
MFVAEMSSDSFQLLGMAERGLLPEFFSKRSRYGTPLFGILFSASGVILLSWLSFQEIVAAEN
FLYCFGMILEFIAFVLLRMKYPHAPRPFKIHGGTVGAILLCIPPTILICVVLALSSFKVMVVS
LAAVAIGLVMQPCLKLIENKRWLKFSISSDLPDDITTHEPLLR*
>Solyc08g005540(SlPut2)
MTVKSETKSSIEVPNAQQQVAVAEKKTSALSSPTTAQNDKFVQNGDDQKVRDLPQKEAAI
PMGECNNAEYIEINEVVSSSRANNDRKLSLLPLVFLIFYEVSGGPFGVEDTVHAAGPLLAL
VGFLVFPIIWSVPEALITAELGTMFPENSGYVVWVSSALGPYWGFQQGWVKWLSGVIDN
ALYPVLFLDYLKSGVPALGGGLPRVLAVIGITLVLTYMNYRGLTIVGWVAVLLGVLSILPFV
VMGLISIPKLKPTRWLATDVHSVDWNLYLNTLFWNLNYWDSISTLVGEVRNPKKTLPKAL
FYAVILVVLSYFFPLLVGTGAVPLERDLWTDGYFSDIAKILGGVWLRWWIQGAAALSNMG
TFVAEMSSDSFQLLGMAERGMLPEFFAKRSRHGTPLVGILLSASGVLLLSWMSFQEIVAAE
NFLYCFGMILEFIAFVRLRIKFPNASRPFKIPGGTVGAIVLCIPPTILVGIVLAFSTVKVMIISL
AAIAIGLVMQPCLKHIEKKKWLKFSISPDLPDIHRDNGTLVH*
>Solyc08g075710(SlPut3)
MVPTTTTTSSSETLQNSSISVADKKTSQQNGDNNNQVLRGVNVGTFKIELHFFMKLRVSA
QREAAIPMGDYNGAEYIGINEVPSPRANNSNKVSLLPLIFLIFYEVSGGPFGVEDTVQAAG
PLFALLGFLIFPLIWSVPEALITAEMGTMFPENGGYVVWVSSALGPYWGFQQGWMKWLS
GVIDNALYPVMFLDYLKSAIPALGGGLPRIVAVLALTVVLTYMNYRGLTIVGWVAVSLGIL
SMLPFVVMGLISIPKIRPERWLVADVHSIDWNLYLNTLFWNLNYWDSISTLAGEVRNPKKT
LPKALFYAVLLVVLSYLFPLLIGTGAVPLERELWTDGYFSDIAKILGGVWLRFWLQGAAAV
SNMGMFVAEMSSDSFQLLGMAERGMLPEFFAKRSRHGTPILGIIFSASGVLLLSWLSFQEIV
AAENFLYCFGMILEFIAFVWLRIKYPNAPRPFKIPGGIIGAILLCVPPAILIGVVLAFSTIKIMI
VSLAAVAIGMVLQPCIKLIERKRWLKFSTSSDLPDITAHGPLIR*
>Solyc01g034080(SlPut4)
MAEEKQTPVSTMAENSEEIPITTAKTVEKSKKLSLIPLIFLIYFEVAGGPYGEEPAVQSAGPL
FAILGFLIFPFIWSVPEALITAELSTTFPGNGGFVTWAYTAFGPFWGSLMGTWKFLSGVINIA
SFPVLCISYMDKLFPIFSSWVPRYMAILVSTLLLSFLNYTGLAIVGYVAVVLGIVSLAPFIVM
SLIAIPKIQPHRWISLGQKGVKKDWNMFFNSLFWNLNFWDNVSTLVGEVENPKRTFPKAL
FSSVILTCFGYLIPLMAVTGAVSVDQREWETGFMANAADMISGKWLKFWIEIGAILSSIGLF
EAQLSTCAFQLLGMAELAFLPKFFALRSKWFNTPWVGILLSTVISSSMSYMNFTDIISSANF
LYSLGMFLELASFLWLRRKYPLINRPYKVPMKMPGLVVMCLIPSVFLAFIMAIATKVVFLIS
GLMTVGGIGWYFFMKLCKTKKWLKFYDDMEEMTIT*

>Solyc10g049640(SlPut5)
MVNDIKKNPKLTLLPLIALIFYDVSGGPFGIEDSVKAGGGPLLSLLGFLIFPLFWSIPEALITA
ELSTSFPQNGGYVIWISSAFGPFWGFQQGFWKWFSGVMDTTLYPLLFLDYLKHSLPIFTHL
IARIPALLTITVSLTYLNYRGLHIVGFSSVLLASFSLLPFLVMGILSIPRINTRKWFVVNFEKV
EWRGYFNNMFWNLNYWDKASTVAGEVDDPSRTFPKALLGAVVLVVLSYIIPLLAGTGAL
DSDSSEWSDGYFAEVGTLIGGLWLKWWIQAAAAMSNMGLFEAEMSSDAYQLLGMSEIG
MLPSVFSSRSKYGTPTISILCSATGTIFLSWMTFQEILEFLNFLYSVGMLLEFAAFINLRIKKP
NLHRPYKVPLQTVGAMLLCLPPSLLLLFVMYLASLKTFIVSGSVIIVGLFLYPAVMYAKEK
QWCHFNTSEQLGLSNDLEDRSSPTELDQIVADDASLSLLGHSKTLRDSKTSSQEISSVD*
>Solyc01g005920(SlPut6)
MGSWKFLTGVINIASFPVLCISYLEEIFPVLDSGVPRKLAILGSTLFLSLVNYTGLTIVGYVA
VALGVISLAPFIIMSLIAIPKIHPHRWLSLGQKGVKKDWNLFFNTLFWNLNFWDNVSTMA
GEVENPRKTFPLALFSSVVFTCLGYIIPLMAVTGAVDVDQRDWDTGFMANAAEMISGKW
LKFWIEIGAVLSTIGLFEAQLSSSAFQILGMAEIAFLPKFFGLRSKWFNTPWVGIVLSTTISL
GMSYMDFQDIISSANFLYSLGMLLEFASFLWLRRKFPLIKRPYRVPMRLPLLVIMCLIPCGF
LVFIMAIATKLVYLISGLMTIGGIGWYFLMNFCKSKKLLKFNDKVDDTYIE*
>Solyc01g111800(SlPut7)
MEAEKYSSLSRGANFTKLSFVPLTFLIFYGVSGGPFGVEDTVRAAGPFLALVGYLIFPIVWS
IPESLITAELSTMFPENGGYVVWVSKSFGPYWGFQLGWVKWMSGVVDNALYPVLFLDYI
KSSVPALANGLPRTIVIVALVIALTYLNYRGLTIVAWVATVLAIFTLLPFLIMGVIALPKLEPS
RWFVMDLENVQWGLYLNTLFWNLNYWDSVSTMAGEVEDPGKTIPKALFYALPLVVSVY
FFPLLFGTGAVPLHRDLWSDGYFSDIAKIIGGVWLRWWVQGASAVSNMGMFLAEMSGDS
YQLLGMAERGMLPEFFAKRSHYGTPFISILFSASGVVLLSCLSFQEIVAAENFMNCFGMILE
FLCFVKLRIKYPAASRPYRIPLGTIGSILICLPPTLFLLVVIALCSFKVMIVSFLAILVGLIMQP
CLIYCDKKKWLSFSVSSDLVELQTNYHQVVEA*
>Solyc09g092420(SlPut8)
MALPSSLFLSTFKSFPLEKQPLEIFNVLIRYYLRVTIISKFMLVCSVLTNDTSKRPLIMFFLYI
YIYIYLDYIKSSVPALANGLPRTIVIVALVIALTYLNYRGLTIVAWVATVLVIFTLLPFLIMGVI
ALPKLEPSRWFVVDLENVQWGLYLNTLFWNLNYWDSVSTMAGEVEDPGKTIPKALFYAL
PLVVSGYFLPLLFGTGAVPLHRDLWSDGYFSDIAKIIGGVWLRLWVQGASAVSNMGMFLA
EMSGDSYQLLGMAERGMLPDFFAKRWRYGTPFISILFSASGVVLLSCLGFQEIVAAENFM
NCFAMILEFLSFVKLRIKYPAASRPYRIPLGSYFSSDKKDMQNRTSQHAKNLLHI*

>Os02g0700500(OsPUT1)
MADTGGRPEVSLATVRSPGHPAASTTAAAAADLGHADTGQEKPTVESAQPANGAAPMGE
CGTEYRGLPDGDAGGPMPSSARTVSMIPLIFLIFYEVSGGPFGIEDSVGAAGPLLAIIGFLVL
PVIWSIPEALITAELGAMFPENGGYVVWVASALGPYWGFQQGWMKWLSGVIDNALYPVL
FLDYLKSGVPALGGGAPRAFAVVGLTAVLTLLNYRGLTVVGWVAICLGVFSLLPFFVMGLI
ALPKLRPARWLVIDLHNVDWNLYLNTLFWNLNYWDSISTLAGEVKNPGKTLPKALFYAVI
FVVVAYLYPLLAGTGAVPLDRGQWTDGYFADIAKLLGGAWLMWWVQSAAALSNMGMF
VAEMSSDSYQLLGMAERGMLPSFFAARSRYGTPLAGILFSASGVLLLSMMSFQEIVAAENF
LYCFGMLLEFVAFILHRVRRPDAARPYRVPLGTAGCVAMLVPPTALIAVVLALSTLKVAVV
SLGAVAMGLVLQPALRFVEKKRWLRFSVNPDLPEIGVIRPPAAPDEPLVP

>Os12g0580400(OsPut2)
MTGACEAAPARRRGLTVLPLVALIFYDVSGGPFGIEDSVRAGGGALLPILGFLVLPVLWSLP
EALVTAELASAFPTNAGYVAWVSAAFGPAAAFLVGFSKWASGTLDNALYPVLFLDYLRSG
GGLVLSPPARSLAVLALTAALTYLNFRGLHLVGLSALALTAFSLSPFVALAVLAAPKIRPSR
WLAVNVAAVEPRAYFNSMFWNLNYWDKASTLAGEVEEPRKTFPKAVFGAVGLVVGAYLI
PLLAGTGALPSETAGEWTDGFFSVVGDRIGGPWLRVWIQAAAAMSNMGLFEAEMSGDSF
QLLGMAEMGMIPAIFARRSRHGTPTYSILCSATGVVILSFMSFQEIVEFLNFLYGLGMLAVF
AAFVKLRVKDPDLPRPYRIPVGAAGAAAMCVPPVVLITTVMCLASARTLVVSAAVAVAGV
AMYYGVEHMKATGCVEFLTPVPPDSLRGSSSSSSSSAASDNGGDDDVEDVCALLLAAGE
HAGEGVSVSKENY
>Os03g0576900(OsPut3)
MVSSHSLLVWIRFLSSFCVWLLGDFLAADADADEDEDAKLRNTAITRANSACLPMEDCV
GIKYSSVNEGEERKGGHGVPKVSIIPLIFLIFYEVSGGPFGIEDSVKAAGPLLAIAGFLLFALI
WSVPEALITAEMGTMFPENGGYVVWVSSALGPFWGFQQGWAKWLSGVIDNALYPVLFL
DYVKSSIPALGGGLPRTLAVLILTVALTYMNYRGLTIVGWVAVFLGVFSLLPFFVMGLIAIPR
IEPSRWLEMDLGNVNWGLYLNTLFWNLNYWDSISTLAGEVENPKRTLPRALSYALVLVV
GGYLYPLITCTAAVPVVREFWTDGYFSDVARILGGFWLHSWLQAAAALSNMGNFVTEMS
SDSYQLLGMAERGMLPEFFAKRSRYGTPLIGIMFSAFGVVLLSWMSFQEIIAAENYLYCFG
MILEFIAFIKLRVVHPNASRPYKIPLGTIGAVLMIIPPTILIVVVMMLASFKVMVVSIMAMLV
GFVLQPALVYVEKRRWLKFSISAELPDLPYSNVEEDSTIPLVC
>Os03g0375900(OsPut4)
MAAEEAHRLADASATAPPKNAAAVDGVAVAAPAAGGGHGRAPGNKLSLVPLIFLIFFEVA
GGPYGAEPAVQSAGPLFALLGFLVFPFIWAVPESLVTAELATAMPGNGGFVLWADRAFGPF
AGGLSIVGWTAVALGVASLSPFALMFGAALPKIRPRRWRATAADKDWKLFFNTLFWNLNY
WDSASTMAGEVERPGRTFPRALLSAVAMTTLGYLLPLLAATGAIDAAPEDWGNGFFADA
AGMIAGGWLKYWIEVGAVLSTIGLYSATLSSAAFQLLGMADLGLLPRAFALRAPVFDTPW
VGILATAAITLAMSFTSFDTIVASANFLYSLGMLLEFAAFVRLRARLPAMPRPYAVPLRGLP
AAAALCAVPSAFLVFVMAIAGWKVYAISAVFTAAGVAVYYLMDLCKARGWLTFSAAAAD
RGGSGGDAMMYRRQGSTASEVV
