# Supplementary material for: Genome-Wide Identification and Characterization of the Polyamine Uptake Transporter (Put) Gene Family in Tomatoes and the Role of Put2 in Response to Salt Stress
Source: Antioxidants (Basel). 2023 Jan 18;12(2):228. doi: 10.3390/antiox12020228 (PMC9952195; doi:10.3390/antiox12020228)
Supplement: Supplementary file 1 [file antioxidants-12-00228-s001.zip › antioxidants-2135816-supplementary/Supplemental data S3.pdf]

Table S1 Primer sequences used for vector construction

Vector	Gene	Forward primers (5'-3')	Reverse primers (5'-3')
**pYES2**	*Put1*	gggaatattaagcttggtaccATGGGGGAGTTCGATGATGG	gcggccgttactagtggatccACGAAGTAAAGGTTCATGTGTTGTAAT
	Put2	gggaatattaagcttggtaccATGACAGTAAAATCAGAAACAAAATCC	gcggccgttactagtggatccTCAATGAACTAATGTTCCATTATCGC
	Put3	gggaatattaagcttggtaccATGACTGAGCTTAGCTCTCCGAA	gcggccgttactagtggatccTTACTCCATCAGGTTTGGTAGGTG
	Put4	gggaatattaagcttggtaccATGGCAGAGGAGAAGCAAACAC	gcggccgttactagtggatccCTATGTTATAGTCATCTCTTCCATATCATCA
	Put5	gggaatattaagcttggtaccATGGTGAATGATATTAAGAAAAACCCA	gcggccgttactagtggatccCTAATCCACTGATGAAATTTCCTGTG
	Put6	gggaatattaagcttggtaccATGACACAAATGATTGAGAC	gcggccgttactagtggatccTTACTCAATATATGTATCAT
	Put7	gggaatattaagcttggtaccATGTTCAATGAAGAAAGAGG	gcggccgttactagtggatccTCAGTCAAGATAAACTTTGATG
	Put8	gggaatattaagcttggtaccATGGGAGTTATCGCGCTTCCT	gcggccgttactagtggatccCTATATATGTAGTAGGTTCT
**pFGC1008-HA**	*Put2*	ttacaattaccatggggcgcgccATGACAGTAAAATCAGAAACAAAATCC	aacatcgtatgggtaggtaccATGAACTAATGTTCCATTATCGC
**CRISPR/Cas9-*put2***	*sgRNA1-Put2*	TCGCCAACCACCTCGTGGGC	TCGCCAACCACCTCGTGGGC
	sgRNA2-Put2	TACTGGGGCTTTCAACAAGG	CCTTGTTGAAAGCCCCAGTA

Table S2 Primer sequences used for qRT-PCR analysis

Gene	Forward primers (5'-3')	Reverse primers (5'-3')
Put1	AGCTGCAGCGTCAAATATGG	CCCTCTCTGCCATACCAAGT
Put2	GGGATCCTCCTCTCAGCTTC	ACACAGTACAATGGCTCCGA
Put3	GGGAATGCTCCCTGAGTTCT	ACACCAGAAGCCGAGAAGAT
Put4	GGCAGAGGAGAAGCAAACAC	CAGATTGAACGGCTGGTTCT
Put5	GCTGCTGCTGCAATGTCTAA	TCCGGTTGCAGAGCATAAGA
Put6	CAACGCGATTGGGATACAGG	TGGTAGACAACACAGCACCA
Put7	ATGCAAACAGGTTGCCGTAG	GACAGACAAGCATGCAGGAA
Put8	TTTCGCAAAGAGATGGCGTT	AAGGTCTTGATGCTGCTGGA
ADC1	CATCCAGTGATTTGCAGCGA	GTAAACCACCCGAAGATGGC
ADC2	GTCGGATATGGCCTTCAGGA	CTTCACGTTCTTCCGGTCAC
SPDS1	AACTCACAGAGCGGGATGAA	AAGAATGCCGAGACACCTCA
SPDS2	TATCTACGCAGGCTGAGAGC	AGGTCCCTCAGTAGAGCAGA
SPDS3	ATGGGCCAGTGTTCCTACAT	AGAGCGCCCTCTAACTTCTC
SPDS4	GCCATCACAATCACCACCAA	ACCTGGCCATAACGCACTAA
ODC1	GCTCAACTCGGAATGCCAAA	CTCAGGGAAGTCGTGGAAGT
ODC2	GGAGCATTGCCGGAAGAAAT	TCGCTTGGCGATAAATGGTG
SPMS1	TATCTACGCAGGCTGAGAGC	AGGTCCCTCAGTAGAGCAGA
SPMS2	TATCTACGCAGGCTGAGAGC	AGGTCCCTCAGTAGAGCAGA
SPMS3	CAATGGCACCATCAACACCA	ACCTTCCCATACGTTGCTGA
PAO1	CCTCTGTGATCATCGTCGGA	CTACTCCGCCGAATTCCTCT
PAO2	TTGCTGCAGATGCTGATTCC	ATCCAAGCGAATGTCAAGCC
PAO3	AGTCTCAAAGGTTGGCGAGA	CAGCAAACCAGCCTTCCATT
PAO4	GCAGAATGGAGGCATGGTTT	CCATCCGTAATCGCCTTCAC
PAO5	GCATGCAGACTCAGCCATCA	ACTTGAGCCAACTGCCACAT
Cu/Zn-SOD	CTCCTGGAGATGAAATCCGT	AAGTGCTCGTCCAACAACTG
MDAR	TCCGAACAAACATACCTGGA	CGTGTGTGCAGTTAGCAATG
DHAR	CCCTGATGTCCTTGGAGACT	AAGAACCATTTGGGCTTGTC
APX	GACTCTTGGAGCCCATTAGG	AGGGTGAAAGGGAACATCAG
GR	TCTGATGCTGCCCTTGA	GCGACTCCTCGGTATGG
CAT1	TGATCGCGAGAAGATACCTG	CTTCCACGTTCATGGACAAC
POD	GATGCAGTTGTGGCTACGGA	GGGCACCTGATAGAAGGACC
GPX	CCGGAACAAATGAGGAGATT	TCCACAAGGAATTTGGTGAA
GAD1	AAACTTCCCATTTCCCAACC	CGATTGATCGGAGGAGAAAA
GAD2	CTTTGATCTTCTCCGTCGTTG	ATATCGAGACGCGAAAGTCG
GAD3	CAGGACGTTTCAATATAATC	CCTACGGAGGGTCTCAGAG
SOS1	TCGAGTGATGATTCTGGTGG	GAGCCTTTCCACACTGTGAT
SOS2	TCTATCCGCTTTGTTTG	ATTGACCAGCCCTATTT
SOS3	CCACCCAAATGCACCAGTAG	CAGCGCCAAAACCATCTCTT
NHX1	GTGGTGTTTGGGCTGAT	TTGCTCGTTAGTGAGAAGTG
NHX2	CCTTTGAGGGGAACAATGG	CATCTTCATCTTCGTCTCC
NHX3	CTCAAGAGTCACCACCAAGCA	CCAACCAAAACAAGACCCAACA
Actin	TGGTCGGAATGGGACAGAAG	CTCAGTCAGGAGAACAGGGT
